# Exercise-induced fatigue and soccer kicking performance: a systematic review and meta-analysis of performance outcomes and contextual moderators

**DOI:** 10.3389/fphys.2026.1818415

**Published:** 2026-06-30

**Authors:** Wenkang Peng, Yecheng Zhang, Dantang Wang, Hugo Sarmento, João Paulo Vilas-Boas, João Ribeiro

**Affiliations:** 1 Centre for Research, Training, Innovation and Intervention in Sport (CIFI2D), Faculty of Sport, University of Porto, Porto, Portugal; 2 Porto Biomechanics Laboratory (LABIOMEP), University of Porto, Porto, Portugal; 3 Facultad de Ciencias de la Actividad Física y del Deporte, Universidad Politécnica de Madrid, Madrid, Spain; 4 College of Physical Education, Shanghai Normal University, Shanghai, China; 5 University of Coimbra, CIPER, Faculty of Sport Sciences and Physical Education, Coimbra, Portugal

**Keywords:** exercise, kinematics, muscle fatigue, shooting performance, sports

## Abstract

**Background:**

The effects of exercise-induced fatigue on soccer kicking performance remain inconsistent across studies and performance metrics, suggesting that fatigue-related impairments may depend on contextual and task-related factors.

**Objective:**

This systematic review and meta-analysis aimed to quantify the effects of exercise-induced fatigue on ball velocity, kicking accuracy, and lower-limb kinematics, and to examine whether selected fatigue protocol, task instruction, and player-level factors explained between-study variability in these effects.

**Methods:**

PubMed, Scopus, SPORTDiscus, and Web of Science were searched from inception to April 2025 and updated in December 2025. Experimental studies in male outfield players with pre-post fatigue comparisons were included. Random-effects meta-analyses (Hedges’ *g*) and predefined subgroup analyses were conducted. Within-participant pre-post correlations were extracted, derived, or imputed, and sensitivity analyses were conducted using alternative correlation assumptions. Risk of bias was assessed using RoBANS-2 and certainty of evidence using GRADE.

**Results:**

Thirty-five studies (733 players) were included in the systematic review, and 31 were meta-analysed. Exercise-induced fatigue was associated with lower ball velocity (Hedges’ *g* = -0.589, 95% CI: -0.789 to -0.388; *p* <.001; *I²* = 72.2%; low certainty) and a smaller reduction in kicking accuracy (*g* = -0.265, 95% CI: -0.449 to -0.081; *p* = .005; *I²* = 58.2%; low certainty). Foot velocity also showed a negative pooled effect (*g* = -0.348, 95% CI: -0.618 to -0.078; *p* = .012), but certainty was very low. Hip, knee, and ankle angular velocities showed no clear pooled changes. Formal subgroup tests suggested moderation by kicking instruction for ball velocity and by fatigue protocol for kicking accuracy; no significant between-subgroup differences were found for player level or the remaining tested subgroup comparisons.

**Conclusions:**

Exercise-induced fatigue was associated with reduced soccer ball velocity and a smaller, less certain reduction in kicking accuracy. Evidence for reduced foot velocity was tentative, whereas evidence for changes in hip, knee, and ankle angular velocities was very uncertain. Contextual variation was partly supported, particularly for kicking instruction and fatigue protocol, but should be interpreted cautiously given heterogeneity and low or very low certainty of evidence.

**Systematic review registration:**

https://osf.io/rmht7/, identifier OSF.IO/RMHT7.

## 1 Introduction

Soccer is a high-intensity, intermittent sport, with high- and maximal-intensity actions superimposed on lower-intensity activity and recovery periods (Hulton et al., 2022). Players repeatedly perform sprints, accelerations, decelerations, changes of direction, sudden stops, jumps, tackles, and physical duels during match play (Mohr et al., 2005). These demands fluctuate across intensity levels and interact with tactical and decision-making constraints. As a result, fatigue can develop after repeated high-intensity efforts and accumulate across match play (Mohr et al., 2005; Silva et al., 2018). Fatigue or impaired performance is commonly observed after short-term intense periods in both halves, at the start of the second half, and toward the end of the game (Mohr et al., 2005). These fatigue-sensitive periods may affect match performance, especially because high-intensity actions are often required in decisive phases of play (Hulton et al., 2022). Heat may further increase physiological strain and contribute to fatigue development during match play (Mohr et al., 2010; Vieira et al., 2023). Therefore, understanding how fatigue affects key technical actions is important for soccer performance.

Exercise-induced physical fatigue in soccer is not explained by a single mechanism. It reflects interacting metabolic, thermal, perceptual, and neuromuscular processes (Mohr et al., 2005; Silva et al., 2018). During prolonged or intense soccer activity, players may experience reduced muscle glycogen or blood glucose availability, lactate accumulation, increased body temperature, dehydration, higher perceived exertion, and reduced force-production capacity (Mohr et al., 2010; Boukhris et al., 2022; Mohr et al., 2023; Vigh-Larsen et al., 2025). These responses may impair repeated high-intensity performance and alter soccer-specific technical execution (Russell et al., 2011; Izquierdo et al., 2020).

Kicking is considered a key soccer-specific skill (Kellis et al., 2006). Its effectiveness depends on the balance between ball velocity and accuracy (Hunter et al., 2018). This balance affects scoring efficiency and overall attacking quality (Ferraz et al., 2017). A higher ball velocity can significantly reduce the reaction time of goalkeepers and defenders. Foot velocity, shank angular velocity, and the transfer of distal velocity to the ball are key factors that determine ball velocity. Among them, foot velocity shows the strongest correlation with ball velocity (Vieira et al., 2018). Beyond shooting, other kicking actions such as passing also rely on coordinated lower-limb mechanics and contribute to ball progression and spatial exploitation during play (Maly et al., 2018).

Current evidence suggests that fatigue can impair kicking performance; however, the type and extent of this effect are not consistent. At the outcome level, reduced ball velocity is the most common finding (Apriantono et al., 2006; Kellis et al., 2006; Ferraz et al., 2012). Changes in accuracy are more complex. Some studies report a decline or show a negative shift in the speed–accuracy balance (Russell et al., 2011). Other studies find only small or non-significant changes (Ferraz et al., 2017). This inconsistency may be partly explained by the source and degree of fatigue. Ball velocity and accuracy will likely decline when intensity exceeds the second lactate threshold (Radman et al., 2016). In contrast, moderate aerobic loads may not affect maximum ball velocity or lower-limb mechanics (Juárez et al., 2011).

Different fatigue protocols may also produce different performance and biomechanical responses. Local resistance or isokinetic exercise directly affect components of the kinetic chain, reducing distal velocity and ball contact quality (Apriantono et al., 2006). Match play, simulated games, or high-intensity interval sessions may be influenced by protocol design and competitive pressure (Stevenson et al., 2017; Izquierdo et al., 2020; Vieira et al., 2023). Mechanistically, fatigue may reduce force-production capacity, alter joint angular velocity, and disrupt proximal-to-distal sequencing and energy transfer. It also reduces the stability of the support leg and ball contact, compromising both speed and accuracy (Apriantono et al., 2006; Kellis et al., 2006; Maly et al., 2018).

Although several reviews have examined the effects of physical fatigue on soccer performance, evidence specifically addressing kicking performance and its biomechanical determinants remains fragmented and inconsistently interpreted (Palucci Vieira et al., 2021; Dambroz et al., 2022). Previous syntheses have typically considered technical performance as a broad construct, with limited differentiation between shooting, passing, dribbling, and other soccer-specific skills, despite the specific speed-accuracy trade-off and kinetic demands of the kicking action (Dambroz et al., 2022). This broad grouping may obscure kicking-specific fatigue effects, because kicking has distinct mechanical and task demands, including the need to balance ball velocity and accuracy (Maly et al., 2018). Moreover, it remains unclear whether different fatigue protocols, task instructions, and competitive levels explain between-study variability in fatigue-related changes in kicking performance. These gaps limit the interpretation of single-study findings and make it difficult to identify the conditions under which fatigue is most likely to impair kicking performance. Therefore, this systematic review and meta-analysis aimed to estimate the effects of exercise-induced physical fatigue on soccer kicking performance and lower-limb kinematics, and to examine whether fatigue protocol, kicking instruction, and player level explain between-study variability.

## 2 Methods

This systematic review with meta-analysis was conducted according to the Preferred Reporting Items for Systematic Reviews and Meta-Analyses (PRISMA) statement (Page et al., 2021). The protocol was registered in the Open Science Framework (OSF) (Registration: https://doi.org/10.17605/OSF.IO/RMHT7).

### 2.1 Eligibility criteria

Based on the Population, Intervention, Comparison, Outcome, and Study Design (PICOS) framework (Methley et al., 2014), predefined inclusion and exclusion criteria were used to determine study eligibility. The target population consisted of male soccer players of any training status, as defined by McKay et al. (2022), provided they were free from chronic disease and recruited as outfield players. Studies exclusively involving goalkeepers or special populations (e.g. players with cerebral palsy or amputees) were not eligible. No restrictions were placed on age.

For the intervention, studies had to include an exercise-induced physical fatigue protocol. Fatigue could be elicited by soccer-specific drills, simulated match play, graded endurance tests, or local muscle loading, with no *a priori* restrictions on intensity, duration, or exercise mode, provided that the protocol was clearly described and documented. Studies were excluded if they did not implement a fatigue intervention, if fatigue was purely mental, or if they focused on test validation rather than fatigue effects.

The comparison criterion required within-participant comparisons of kicking performance before and after an exercise-induced physical fatigue protocol. Official-match studies were eligible only when they included a standardized pre-post kicking test; studies reporting only match-derived statistics without a standardized kicking test were excluded.

Regarding outcomes, studies were eligible if they reported at least one measure of kicking performance (ball velocity or accuracy) or any lower-limb kinematic variable related to the kicking action. Studies focusing exclusively on other technical skills (e.g. passing without shooting) or not involving a ball-kicking action were excluded.

For study design, only original peer-reviewed articles with a controlled experimental or quasi-experimental design (parallel or crossover, randomized or non-randomized) and available full text in English were included. Conference abstracts, theses and dissertations, books or book chapters, review articles, case reports, acute single-bout studies without a fatigue protocol, and animal studies were excluded. No publication year limits were imposed. No additional language restriction was applied beyond the requirement for full-text availability in English.

### 2.2 Databases and search strategies

Literature searches were conducted across multiple electronic databases, including PubMed, Scopus, SPORTDiscus, and Web of Science, to ensure comprehensive coverage of sports science and soccer-related research. The terms used for the searches were: (soccer OR football* OR “association football”) AND (fatigu* OR exercis* OR exhausti* OR “match demands” OR “post-match” OR “match-related fatigue”) AND (kick* OR shoot* OR skill* OR technical) AND (biomechanic* OR kinematic* OR “motion analysis” OR “3D motion” OR velocity OR speed OR accuracy OR precision OR performance). These descriptors were combined using the Boolean operators “OR” and “AND”. Articles were considered from their date of publication through April 2025 and were updated in December 2025. The full search strategies for all databases are provided in **Supplementary Tables S2** and **S3**.

### 2.3 Study selection

Two authors (WP and DW) independently performed the initial screening. Duplicates were first identified and removed using Zotero reference management software (Zotero 7), which was also used to organize records throughout the screening process. After removing duplicates, the titles and abstracts were screened to determine potentially eligible studies. Subsequently, the full-text studies were examined using the inclusion and exclusion criteria to determine the final included literature. In the event of disagreement, a third author (YZ) resolved the differences and determined whether the conflicting studies were included or excluded.

### 2.4 Data extraction

Data were extracted from each included study, capturing the following key information: author names, publication year, research objectives, sample description (age, number, and level of the subjects), detailed description of the fatigue induction protocol, fatigue quantification metrics (heart rate, blood lactate concentrations, and rating of perceived exertion), kicking test characteristics (trials, interval, instruction, target, and approach), and results (ball velocity, accuracy, and kinematic variables). Two authors (WP and DW) independently performed data extraction, with discrepancies resolved through discussion or adjudication by a third author (YZ). Extracted data were compiled into an Excel spreadsheet for subsequent analysis. The authors were contacted to request the necessary information if data were missing or presented only graphically. If this was unsuccessful and the data remained in graphical form, relevant data were extracted using WebPlotDigitizer 5 (https://automeris.io/WebPlotDigitizer). A validation check showed high agreement with original data (*r* = 0.99, *p* < 0.001) (Drevon et al., 2017). Studies for which missing data could not be obtained were excluded from the final analysis. For each meta-analysed outcome, pre- and post-fatigue means, standard deviations, and sample sizes were extracted. When several eligible contrasts were available within the same study, one effect was selected for each meta-analytic outcome to reduce dependency. We prioritized the contrast that best represented the main fatigue effect, including the fatigued condition over control or recovery conditions, the highest or final fatigue stage over intermediate stages, the placebo or usual-practice condition over supplement/recovery interventions, the dominant/preferred limb over the non-dominant limb when both were reported, and the standardized outcome most directly matching the meta-analysed domain. Study-specific decisions are reported in **Supplementary Table S5**.

### 2.5 Risk of bias and methodological reporting quality

Risk of bias and methodological reporting quality assessments were conducted independently by two reviewers (WP and DW). A third reviewer (YZ) resolved any disagreements through discussion. The risk of bias was assessed using the Risk of Bias Assessment Tool for Non-randomized Studies (RoBANS-2) (Seo et al., 2023), which evaluates the comparability of the target group, target group selection, confounders, measurement of exposure, blinding of assessors, outcome assessment, incomplete outcome data, and selective outcome reporting. Methodological reporting quality was assessed descriptively using the 9 questions (Q1-9) from the checklist presented by Palucci Vieira et al. (2021) (Palucci Vieira et al., 2021). The sum of scores from all questions was computed (0-18) and converted into percentages (0-100%). Studies were classified as having high (>=75%), moderate (50-74%), or low (<50%) methodological reporting quality (Palucci Vieira et al., 2019). This checklist was used only to describe methodological reporting quality.

### 2.6 Statistical analysis

The meta-analysis used Comprehensive Meta-Analysis (CMA) 3.0 software (Borenstein, 2022). Because all meta-analyzed effects were derived from within-participant pre-post fatigue comparisons, pre- and post-fatigue values were not treated as independent groups. Effect sizes were calculated using the one-group pre-post/matched-groups option in CMA, with means, pre-fatigue SD, post-fatigue SD, pre-post correlation, and sample size entered for each effect.

Due to differences in fatigue protocols, participant characteristics, and kicking-test designs, random-effects models were used for continuous outcomes. Hedges’ *g* was used as the primary effect size. For each study, the effect direction was coded as the post-fatigue mean minus the pre-fatigue mean, so that negative Hedges’ *g* values indicated poorer post-fatigue performance relative to pre-fatigue performance. Hedges’ *g* was calculated in CMA as the standardized mean difference with small-sample correction under the one-group pre-post/matched-groups data structure. For accuracy outcomes expressed as error, deviation, or distance from the target, effect directions were reverse-coded where necessary so that negative values consistently represented poorer accuracy after fatigue. Absolute effect magnitudes were interpreted as trivial (<0.2), small (0.2–0.5), medium (0.5–0.8), and large (>0.8) (Cohen, 1988).

For within-participant pre-post designs, the sampling variance and standard error of Hedges’ g incorporated the pre-post correlation. When *r* was reported or could be derived from paired statistics, the study-specific value was used. When the SD of the change score was reported, *r* was calculated from the relationship between the pre-fatigue SD, post-fatigue SD, and change-score SD (Higgins et al., 2019):


$$r=\frac{SD_{pre}^{2}+SD_{post}^{2}-SD_{change}^{2}}{2\times SD_{pre}\times SD_{post}}$$

When confidence intervals for paired mean changes were reported, the SD of change was first derived from the standard error of the change score. If r could not be derived, *r* = 0.50 was used in the primary analysis, and sensitivity analyses were conducted using *r* = 0.30 and *r* = 0.70. Details are provided in **Supplementary Tables S5** and **S6**.

Given the wide range of kinematic outcomes reported across studies, a pragmatic criterion was adopted for quantitative synthesis. Only kinematic variables reported by at least two independent studies with reported or derivable pre- and post-fatigue means, standard deviations, and sample sizes were included in the meta-analysis (Stojanović et al., 2020). Consequently, pooled kinematic analyses were restricted to the linear velocity of the kicking foot and the angular velocities of the hip, knee, and ankle.

The *I²* statistic was used to quantify between-study heterogeneity (Nakagawa et al., 2017). The following interpretations were applied: <25%, might not be important; 25%-50%, may represent moderate heterogeneity; >50%-75%, may represent substantial heterogeneity; and >75%-100%, considerable heterogeneity (Cumpston et al., 2019). Alongside 95% CIs, 95% prediction intervals (PIs) were calculated to reflect the expected range of effects in future similar studies, following recommendations that PIs provide a more realistic estimate of between-study heterogeneity (Borg et al., 2024).

Publication bias was assessed using Egger’s regression only for outcomes with at least 10 studies (Sterne et al., 2011). For outcomes with fewer than 10 studies, publication bias was considered not reliably assessable. Leave-one-out sensitivity analyses were performed to assess the influence of each study on the overall effect size and to test the robustness of the findings (Tobias, 1999). Certainty of evidence for each pooled outcome was assessed using the GRADE framework, considering risk of bias, inconsistency, indirectness, imprecision, and publication bias (Schünemann et al., 2019). Evidence was classified as high, moderate, low, or very low certainty. GRADE assessments were completed by one reviewer (WP) and verified by a second reviewer (DW), with disagreements resolved through discussion. Downgrading decisions are detailed in **Supplementary Tables S8** and **S9**. Statistical significance was set at p <.05.

### 2.7 Subgroup classification strategy

To explore potential sources of heterogeneity and examine whether study characteristics moderated the effects of fatigue on kicking performance, predefined subgroup analyses were conducted. The following moderators were considered: fatigue protocol type; competitive level of the players; and kicking instruction.

Fatigue protocols were grouped into four categories: (i) simulated match demand (SMD), comprising match-play exposures and match-simulation protocols that imposed general intermittent endurance demands. Official-match studies were classified as SMD only when a standardized pre-post kicking test was administered; (ii) soccer-specific exercise (SSE), consisting of intermittent, soccer-specific high-intensity drills; (iii) graded exhaustion (GE), including incremental treadmill, cycle-ergometer, Yo-Yo, or shuttle-running tests performed until volitional exhaustion or the highest workload; and (iv) local muscle fatigue (LMF), involving lower-limb resistance or isolated muscular loading designed to induce peripheral neuromuscular fatigue. Player level was coded using the competitive label reported in each study. For subgroup analyses, professional, semi-professional, elite, and elite-academy samples were collapsed into the professional category; amateur and university samples were collapsed into the amateur category; youth and elite-youth samples, as well as samples with players younger than 18 years, were collapsed into the youth category. Studies that could not be classified unambiguously were excluded from the relevant subgroup analysis, as detailed in **Supplementary Table S7**. Kicking instructions were classified into three categories: (i) maximal ball-velocity shots without an explicit target-accuracy requirement; (ii) target-accuracy shots without an explicit speed requirement; and (iii) target-accuracy shots while simultaneously aiming for high or maximal ball velocity. Between-subgroup differences were evaluated using Q-between statistics. Subgroup-specific significance was not interpreted as evidence of moderation unless the Q-between test was significant.

## 3 Results

### 3.1 Study identification and selection

**Figure 1:**
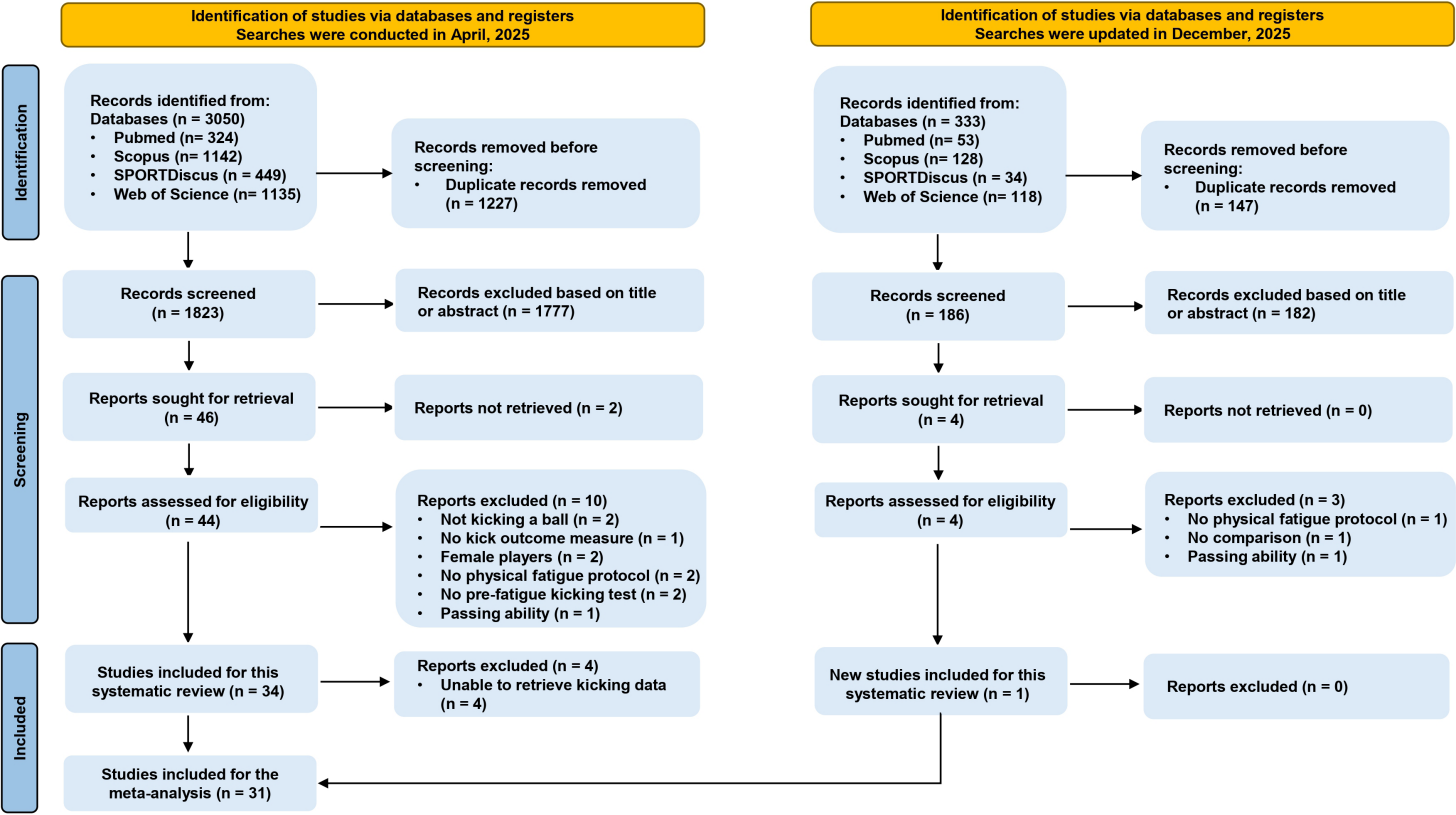
PRISMA 2020 flow diagram of study identification, screening, eligibility assessment, and inclusion.

The search process resulted in 3383 studies: 3050 from the primary search, and 333 from an updated search conducted 8 months later. After screening, 35 studies were deemed eligible to include in the systematic review, of which 31 studies were included in the meta-analysis, as illustrated in **Figure 1**.

### 3.2 Methodological reporting quality and risk of bias

**Figure 2:**
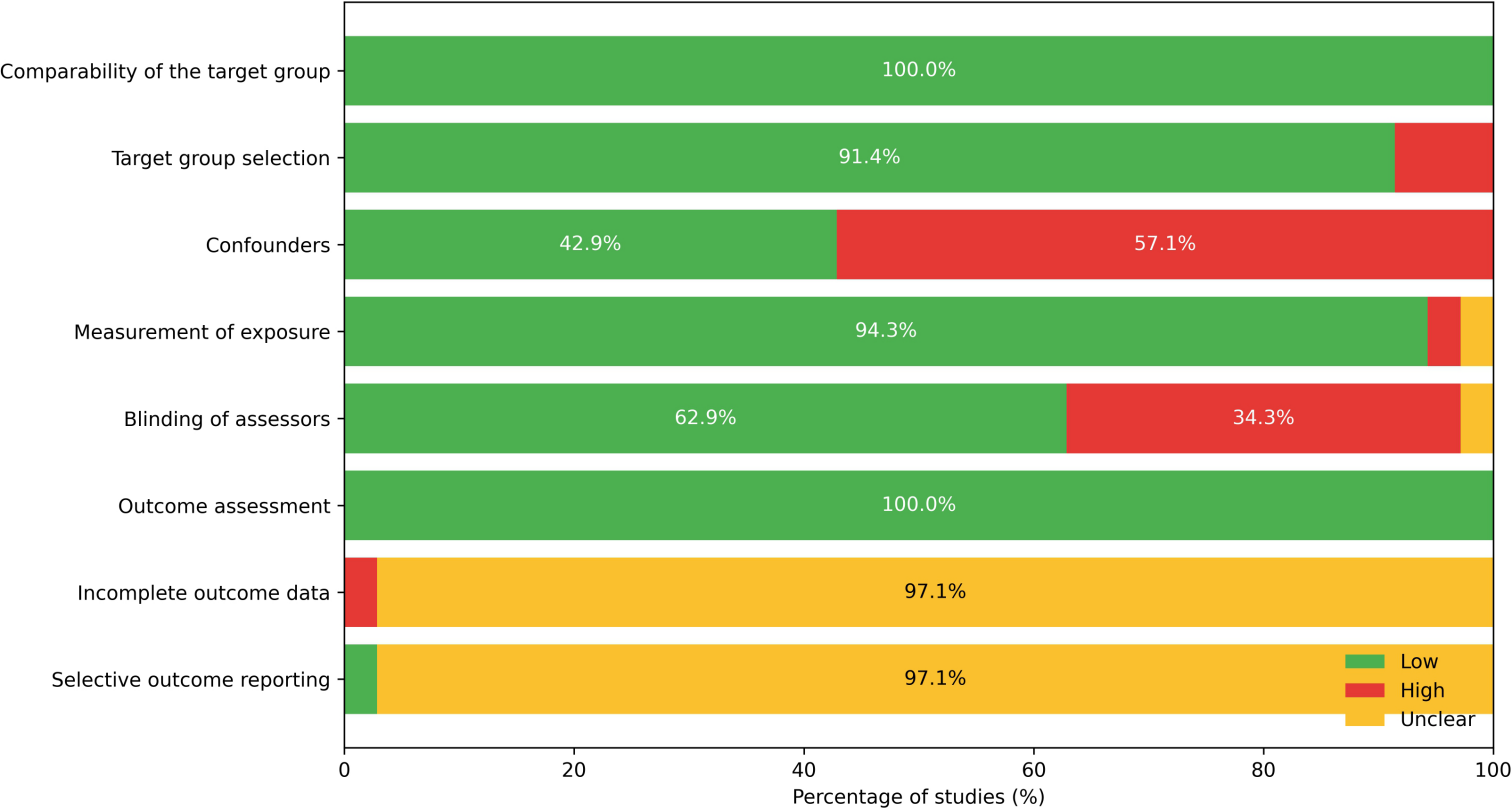
Risk-of-bias summary across the included studies assessed using RoBANS-2. Bars represent the percentage of studies judged as low, high, or unclear risk of bias for each domain.

The 35 studies included in the systematic review had a mean methodological reporting quality score of 77 ± 10%. Among them, 60% scored above 75% and were therefore considered of acceptable reporting quality. The lowest and highest mean scores were observed for Q4 “Ball standardization” (0.88 ± 0.59 points) and Q2 “Included players were characterized” (2 points), respectively. Risks of bias for each key criterion are presented as percentages across the literature in **Figure 2**, and study-level judgments are provided in **Supplementary Figure 1**; all 35 studies were evaluated. The highest risk of bias was found in the “Confounders” domain, with 57% of studies rated as having high risk of bias. The “Blinding of assessment” domain followed, with 34% of studies judged to be at high risk. The greatest uncertainties were observed in “Selective outcome reporting”, where 97% of studies were rated as unclear, and in “Incomplete outcome data”, where 97% of studies were rated as unclear. By contrast, the remaining domains showed relatively low risk of bias. All studies were rated as low risk in the “Comparability of the target group” domain. In the “Target group selection” domain, 91% of studies were rated as low risk, and in the “Measurement of exposure” domain, 94% of studies were rated as low risk.

### 3.3 Study characteristics

#### 3.3.1 General information

The included studies assessed 733 players across professional, semi-professional, amateur, university, elite-youth, and youth samples. Detailed study characteristics are presented in **Supplementary Table S1**, and study-level player classifications are presented in **Supplementary Table S7**. Most studies were conducted on soccer fields (47%), in laboratory environments (15%), or in indoor facilities (15%), while 23% of studies did not specify the experimental setting.

#### 3.3.2 Experimental methods

Regarding the kicking tests, 34% of studies examined shots from a stationary ball, 14% from a rolling ball, and two studies assessed both conditions. The remaining 46% of studies did not provide details. The instep kick was the most frequently analyzed technique (37%). Only one study explicitly reported the use of the inside kick, while 60% of studies did not specify the part of the foot used during the kick. Regarding the approach, approximately 29% of studies reported at least one characteristic of the run-up, whereas 71% did not provide this information or allowed participants to choose freely. Instructions given to participants varied. In 11% of studies, players were instructed to kick the ball at maximum speed without concern for accuracy. In 20% of the cases, players were asked to kick the ball at maximum speed toward the goal. In 26%, players were instructed to hit a target regardless of ball velocity. In 14%, they were instructed to kick at maximum speed and hit a target. In 11%, the instruction was to hit the target while maintaining maximum speed. Two studies included both maximum-speed shots and target-hitting tasks. In addition, 11% of studies did not report the instructions provided. The target configurations also varied. In 29% of studies, players aimed at the entire goal. In 22%, they aimed only at the center of the goal. In 17%, they aimed at a specific target within the goal. In 11% of cases, the target was limited to the four corners of the goal. The remaining 20% of studies did not report target configurations. Information on the number of trials, sets, and recovery intervals is provided in **Supplementary Table S1**.

#### 3.3.3 Data collection

A total of 60% of the studies measured ball velocity during kicking, most often using radar guns or three-dimensional (3D) motion analysis systems. Accuracy was assessed in 57% of the studies; about half of these studies used notational or score-based measures, such as the number of goals, success rates, hit rates, shooting scores, or the number of kicks that hit a designated target. The other half calculated the deviation or distance of the ball relative to the target to quantify accuracy-related performance. Kinematic variables during kicking were reported in 29% of the studies, primarily including the linear velocity of the kicking foot and angular velocities of the ankle, knee, and hip joints. Common indicators of physical fatigue were also assessed. Then, 37% of studies measured perceived exertion ratings (RPE), 43% measured blood lactate concentration, and 46% monitored heart rate.

### 3.4 Primary analysis

**Figure 3:**
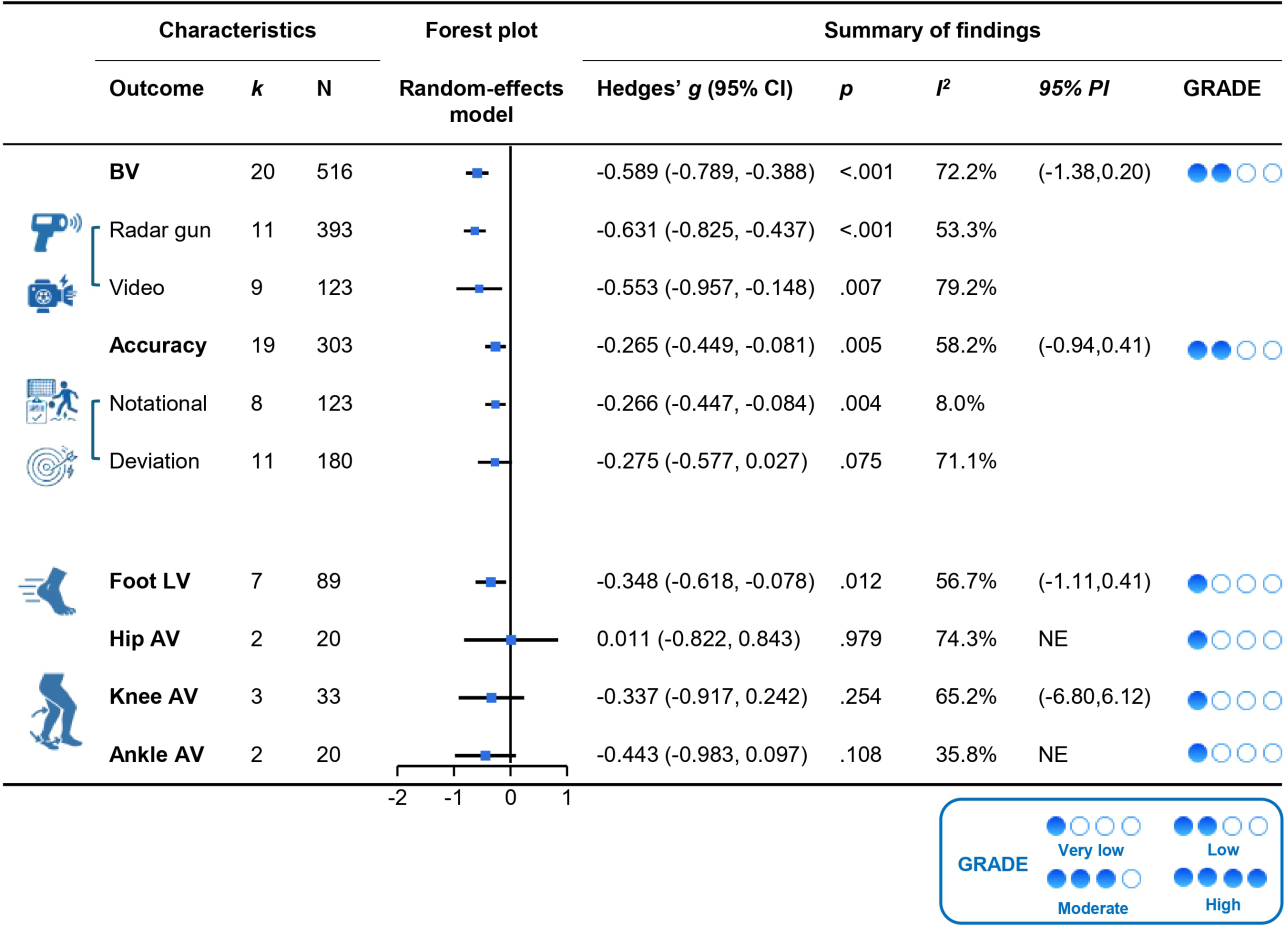
Summary of findings for the effects of exercise-induced fatigue on soccer kicking performance and lower-limb kinematics. BV, ball velocity; Acc, accuracy; Foot LV, foot linear velocity; Hip AV, hip angular velocity; Knee AV, knee angular velocity; Ankle AV, ankle angular velocity; CI, confidence interval; PI, prediction interval; GRADE, Grading of Recommendations Assessment, Development and Evaluation.

This meta-analysis focused on ball velocity, kicking accuracy, foot velocity, and the angular velocities of the hip, knee, and ankle joints during kicking. Effect sizes were coded so that negative values indicate poorer post-fatigue performance. Summary-of-findings data are summarized in **Figure 3** in the main manuscript, and the complete Summary of Findings table is provided in **Supplementary Table S8**. Domain-level GRADE judgments and downgrading reasons are provided in **Supplementary Table S9**. Sensitivity analyses using alternative imputed pre-post correlations (*r* = 0.30 and *r* = 0.70) did not change the direction or statistical interpretation of the pooled effects for ball velocity, kicking accuracy, or foot velocity (**Supplementary Table S6**). Publication-bias testing was conducted only for ball velocity and kicking accuracy, as these were the only outcomes with at least 10 studies.

#### 3.4.1 Ball velocity

Fatigue significantly reduced ball velocity compared with the pre-fatigue condition (*k* = 20, *g* = -0.589, 95% CI: -0.789 to -0.388, *p* <.001, *I²* = 72.2%, 95% PI: -1.38 to 0.20; low certainty). Egger’s regression did not indicate significant small-study effects for ball velocity. Leave-one-out analyses confirmed that the pooled effect remained statistically significant, indicating that the overall result was not driven by a single study.

#### 3.4.2 Accuracy

Fatigue significantly reduced kicking accuracy compared with the pre-fatigue condition (*k* = 19, g = -0.265, 95% CI: -0.449 to -0.081, *p* = .005, *I²* = 58.2%, 95% PI: -0.94 to 0.41; low certainty). Egger’s regression did not indicate significant small-study effects for kicking accuracy. Leave-one-out analyses indicated that the pooled effect remained statistically significant, suggesting that the result was not driven by any single study.

#### 3.4.3 Foot velocity

Foot velocity was also reduced on average after fatigue (*k* = 7, *g* = -0.348, 95% CI: -0.618 to -0.078, *p* = .012, *I²* = 56.7%, 95% PI: -1.11 to 0.41), but the certainty of evidence was very low. Publication bias was not reliably assessable because fewer than 10 studies were available. Leave-one-out analyses indicated instability: removal of several individual studies rendered the pooled effect non-significant. Therefore, the evidence for reduced foot velocity should be interpreted as tentative.

#### 3.4.4 Hip, knee, and ankle joint angular velocity

The angular velocities of the hip (*k* = 2, *g* = 0.011, 95% CI: -0.822 to 0.843, *p* = .979, *I²* = 74.3%, 95% PI: not estimable; very low certainty), knee (*k* = 3, *g* = -0.337, 95% CI: -0.917 to 0.242, *p* = .254, *I²* = 65.2%, 95% PI: -6.80 to 6.12; very low certainty), and ankle (*k* = 2, *g* = -0.443, 95% CI: -0.983 to 0.097, *p* = .108, *I²* = 35.8%, 95% PI: not estimable; very low certainty) showed no clear pooled changes after fatigue. Publication bias was not reliably assessable for these outcomes because fewer than 10 studies were available.

### 3.5 Moderator analysis

Moderator analyses were conducted to examine whether player level, fatigue protocol, and kicking instruction explained between-study differences in ball velocity and kicking accuracy. Formal between-subgroup differences were evaluated using Q-between statistics; subgroup-specific significance alone was not interpreted as evidence of moderation.

#### 3.5.1 Potential moderators of ball velocity

**Figure 4:**
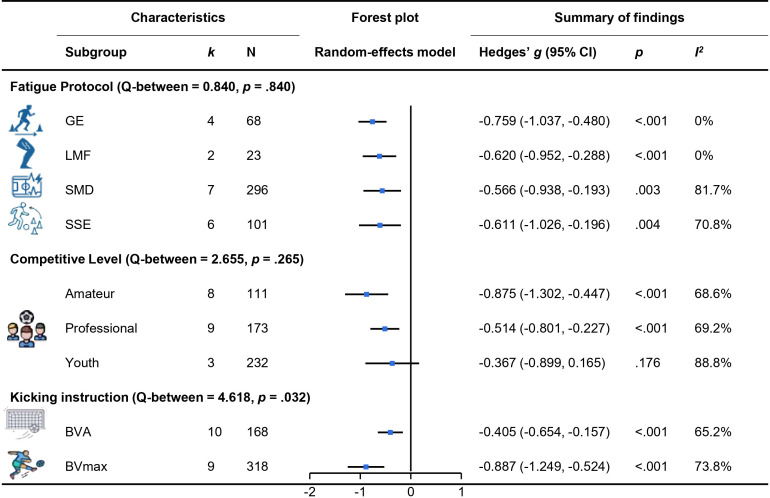
Subgroup meta-analysis of the effects of exercise-induced fatigue on ball velocity according to fatigue protocol, competitive level, and kicking instruction. Values are Hedges’ g with 95% confidence intervals.

For ball velocity, only kicking instruction showed a significant between-subgroup difference (*Q* = 4.618, *p* = .032). Fatigue protocol (*Q* = 0.840, *p* = .840) and player level (*Q* = 2.655, *p* = .265) did not show statistically significant between-subgroup differences. By fatigue protocol, negative pooled effects were observed for GE (*k* = 4, *g* = -0.759, 95% CI: -1.037 to -0.480, *p* <.001, *I²* = 0%), LMF (*k* = 2, *g* = -0.620, 95% CI: -0.952 to -0.288, *p* <.001, *I²* = 0%), SMD (*k* = 7, *g* = -0.566, 95% CI: -0.938 to -0.193, *p* = .003, *I²* = 81.7%), and SSE (*k* = 6, *g* = -0.611, 95% CI: -1.026 to -0.196, *p* = .004, *I²* = 70.8%). By player level, pooled effects were negative for amateurs (*k* = 8, *g* = -0.875, 95% CI: -1.302 to -0.447, *p* <.001, *I²* = 68.6%), professionals (*k* = 9, *g* = -0.514, 95% CI: -0.801 to -0.227, *p* <.001, *I²* = 69.2%), and youth players (*k* = 3, *g* = -0.367, 95% CI: -0.899 to 0.165, *p* = .176, *I²* = 88.8%). By instruction, the effect was larger for maximal ball-velocity instructions (BVmax: *k* = 9, *g* = -0.887, 95% CI: -1.249 to -0.524, *p* <.001, *I²* = 73.8%) than for combined ball-velocity and accuracy instructions (BVA: *k* = 10, *g* = -0.405, 95% CI: -0.654 to -0.157, *p* <.001, *I²* = 65.2%). The subgroup meta-analysis results for ball velocity are summarized in **Figure 4**.

#### 3.5.2 Potential moderators of accuracy

**Figure 5:**
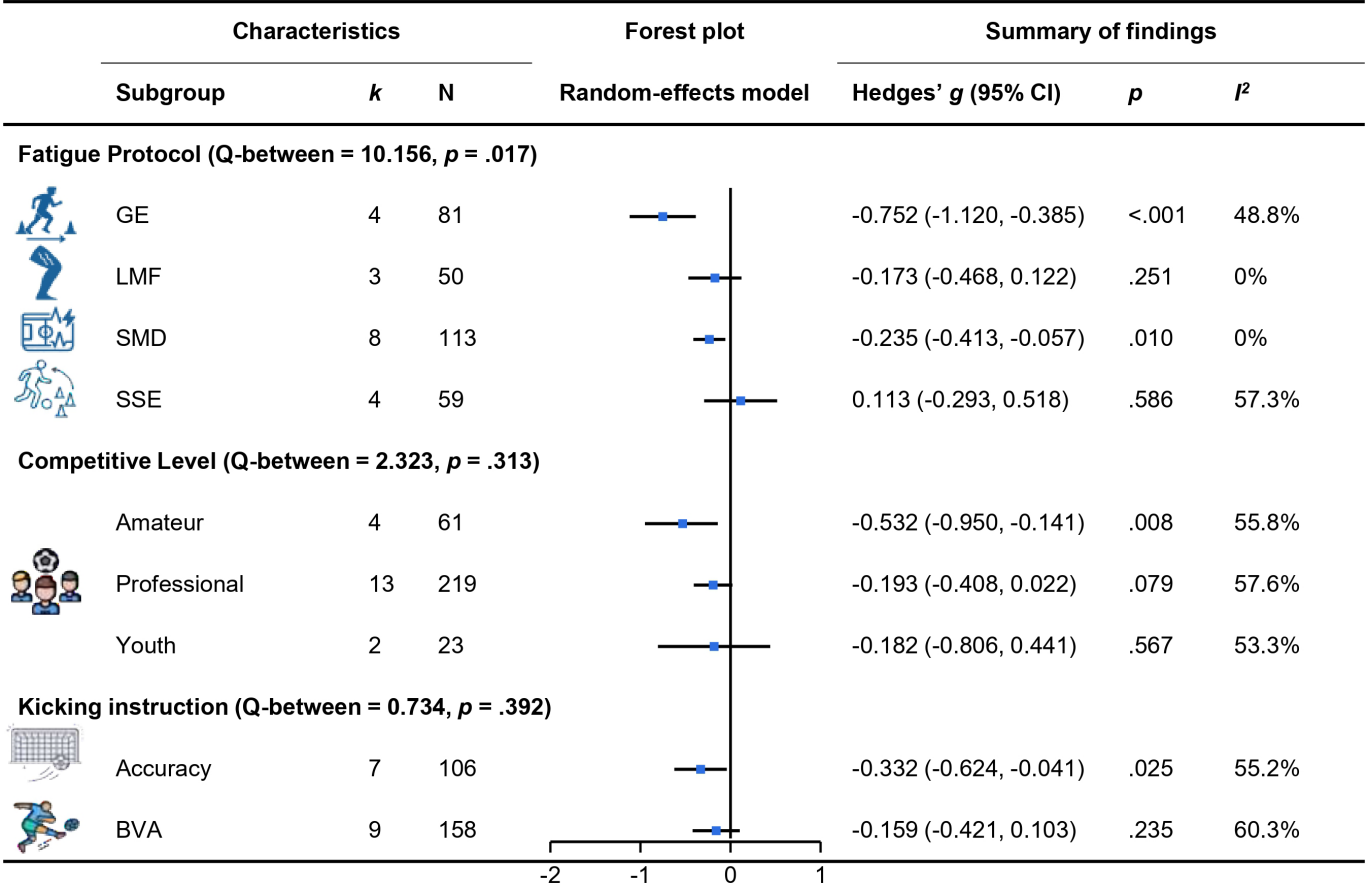
Subgroup meta-analysis of the effects of exercise-induced fatigue on kicking accuracy according to fatigue protocol, competitive level, and kicking instruction. Values are Hedges’ g with 95% confidence intervals.

For kicking accuracy, only fatigue protocol showed a significant between-subgroup difference (*Q* = 10.156, *p* = .017). Player level (*Q* = 2.323, *p* = .313) and kicking instruction (*Q* = 0.734, *p* = .392) did not show significant between-subgroup differences. By fatigue protocol, GE showed the largest negative pooled effect (*k* = 4, *g* = -0.752, 95% CI: -1.120 to -0.385, *p* <.001, *I²* = 48.8%), followed by SMD (*k* = 8, *g* = -0.235, 95% CI: -0.413 to -0.057, *p* = .010, *I²* = 0%). LMF showed no clear effect (*k* = 3, *g* = -0.173, 95% CI: -0.468 to 0.122, *p* = .251, *I²* = 0%), and SSE showed no negative pooled effect (*k* = 4, *g* = 0.113, 95% CI: -0.293 to 0.518, *p* = .586, *I²* = 57.3%). By player level, pooled effects were negative for amateurs (*k* = 4, *g* = -0.532, 95% CI: -0.950 to -0.141, *p* = .008, *I²* = 55.8%), professionals (*k* = 13, *g* = -0.193, 95% CI: -0.408 to 0.022, *p* = .079, *I²* = 57.6%), and youth players (*k* = 2, *g* = -0.182, 95% CI: -0.806 to 0.441, *p* = .567, *I²* = 53.3%). By instruction, accuracy-only tasks showed a negative pooled effect (*k* = 7, *g* = -0.332, 95% CI: -0.624 to -0.041, *p* = .025, *I²* = 55.2%), whereas combined ball-velocity and accuracy tasks did not show a clear effect (BVA: *k* = 9, *g* = -0.159, 95% CI: -0.421 to 0.103, *p* = .235, *I²* = 60.3%). The subgroup meta-analysis results for kicking accuracy are summarized in **Figure 5**.

## 4 Discussion

The present synthesis does not support a uniform impairment of soccer kicking performance after exercise-induced fatigue. Instead, the pooled evidence indicates a selective pattern of impairment. The strongest evidence was for a reduction in ball velocity, although certainty was low because of risk of bias and substantial heterogeneity. Kicking accuracy also showed a smaller average reduction with low certainty. Evidence for reduced foot velocity was tentative, whereas evidence for changes in hip, knee, and ankle angular velocities was very uncertain. Moreover, the prediction intervals for ball velocity, kicking accuracy, and foot velocity crossed zero, indicating that these average effects should not be expected uniformly across all study contexts. Formal subgroup tests supported moderation by kicking instruction for ball velocity and by fatigue protocol for accuracy. Other contextual factors, including player level, were not supported by significant between-subgroup tests.

### 4.1 The impact of exercise-induced fatigue on kicking performance

#### 4.1.1 Ball velocity

Ball velocity decreased across fatigue-protocol subgroups, but the magnitude of decline did not differ significantly by protocol type. The larger reduction under maximal ball-velocity instructions suggests that task instruction affects how fatigue is expressed in ball velocity.

Under LMF and SSE conditions, velocity loss may partly reflect neuromuscular constraints. LMF protocols directly fatigue the knee extensors and ankle musculature, reducing distal segment velocity and impairing foot–ball contact quality (Apriantono et al., 2006; Carstensen et al., 2024). Decreases in foot linear velocity, peak shank angular velocity, and proximal-to-distal energy transfer efficiency weaken the whip-like coordination necessary for high ball velocity (Apriantono et al., 2006). Studies employing quantifiable fatigue induction suggest that declines in neuromuscular function may be associated with ball-velocity loss, whereas accuracy is not necessarily affected (Carstensen et al., 2024). In contrast, SSE protocols introduce greater contextual variability. Ball velocity tends to decline predominantly under high-intensity loads, whereas low-to-moderate intensities produce minimal changes (Ferraz et al., 2017). Environmental stressors, such as heat exposure, amplify decrements, whereas recovery interventions (e.g., cooling) may partially attenuate velocity loss (Vieira et al., 2023). The greater heterogeneity observed in SSE likely reflects this task and context dependency.

In GE and SMD protocols, ball-velocity loss may reflect endurance-related fatigue, although the temporal expression of impairment may differ between conditions. GE progressively increases exercise intensity to exhaustion, rapidly disrupting metabolic homeostasis through high-energy phosphate depletion and acid–base disturbances, consistent with the critical power framework (Poole and Barstow, 2015). Studies defining intensity relative to physiological thresholds demonstrate a threshold-type response, with kicking performance largely preserved at low-to-moderate intensities but declining once exercise exceeds the second lactate threshold (Radman et al., 2016). These impairments are typically transient, as ball velocity and lower-limb linear velocities recover rapidly within the first post-exhaustion attempts (Katis et al., 2014). By contrast, SMD protocols replicate prolonged intermittent match demands, resulting in a progressive, time-dependent decline in ball velocity that becomes more pronounced in the later stages of play (Izquierdo et al., 2020). This pattern is accompanied by reductions in shank angular velocity and joint moment generation, indicating increasing constraints on force production and inter-segmental power transfer (Kellis et al., 2006). Thus, GE may represent a more acute and reversible impairment, whereas SMD may reflect more cumulative mechanical constraints.

Task instructions also influenced the effects on ball velocity. Fatigue had the largest impact when instructions emphasized “maximum speed only” (Amiri-Khorasani et al., 2011; Mor et al., 2022), and smaller when accuracy constraints were introduced (Russell et al., 2012). These trends align with the speed–accuracy trade-off. When a target is introduced, players may reduce force generation and foot velocity to preserve placement stability, and this strategy becomes more pronounced under fatigue. Therefore, part of the “apparent” velocity decline reflects behavioral prioritization rather than pure physical limitation (Stone and Oliver, 2009; Russell et al., 2011). Task priorities determine how strongly fatigue manifests on the ball-velocity outcome.

Ball velocity decreased in both amateur and professional subgroups, whereas the youth subgroup showed no clear pooled effect. The reduction was numerically larger in amateur players, but the between-subgroup test was not significant. Therefore, this result does not confirm a player-level effect. One possible explanation is that high-level players may experience less match-related fatigue and recover faster (Rampinini et al., 2011), whereas less stable kicking conditions may be more sensitive to fatigue (Katis et al., 2017). Further studies are needed to test whether competitive level moderates fatigue-related changes in ball velocity.

#### 4.1.2 Kicking accuracy

Fatigue also reduced kicking accuracy, though the pooled effect was smaller than for ball velocity and certainty was low. Protocol type showed a statistically supported moderation effect for accuracy; differences by kicking instruction and player level were not confirmed by between-subgroup tests.

GE showed the largest pooled reduction in accuracy, particularly when intensity exceeded the second lactate threshold (Radman et al., 2016). Near exhaustion, placement control deteriorates rapidly, with increased deviation and reduced hit frequency (Maly et al., 2018). Under SMD conditions, accuracy tends to deteriorate as intermittent load accumulates over time. After 45 minutes of intermittent match play, the kicking scores decline significantly, also demonstrating a shift in ball placement from optimal to suboptimal zones (Stone and Oliver, 2009). However, extended simulations do not consistently demonstrate progressive deterioration, suggesting that detectability depends on task sensitivity and contextual constraints (Stevenson et al., 2017).

By contrast, LMF and SSE protocols yielded inconsistent accuracy effects. Isolated neuromuscular fatigue may suppress force output without disrupting aiming control (Carstensen et al., 2024). Accuracy declines may be more likely when metabolic strain accompanies local fatigue (Rampinini et al., 2011). Similarly, soccer-specific exercise protocols showed no clear pooled reduction in accuracy, consistent with studies reporting minimal pre–post changes despite varying intensity (Ferraz et al., 2017), although high-intensity work combined with environmental stress may increase radial error (Vieira et al., 2023).

Measurement choices also matter. Notational measures may be less sensitive to subtle fatigue effects, whereas continuous deviation metrics may better capture losses of stability and increased dispersion (Maly et al., 2018). Time pressure amplifies the decline. After 45 minutes of the Loughborough Intermittent Shuttle Test, accuracy falls when shots are time-limited and improves when the time constraint is removed (Stone and Oliver, 2009). Recovery kinetics also differ; accuracy often recovers faster than speed, sometimes approaching baseline within 5 minutes (Vieira et al., 2023).

When instructions prioritize accuracy, fatigue effects may be attenuated. This interpretation is consistent with the speed–accuracy trade-off framework, whereby players may preserve placement at reduced execution speed (Russell et al., 2011; Ferraz et al., 2017). However, kicking instruction did not significantly moderate accuracy. The speed–accuracy trade-off may therefore explain task strategies, but it was not supported as a source of between-subgroup differences. Once exercise intensity exceeds key physiological thresholds, the capacity to maintain accurate ball placement may become increasingly challenged, and accuracy impairments may become more apparent under severe load conditions (Radman et al., 2016).

#### 4.1.3 Kinematic variables

The kinematic findings were less conclusive than the performance outcomes. Foot velocity tended to decrease after fatigue, which is consistent with the reduction in ball velocity, but the evidence was not strong enough to treat this as a robust biomechanical finding. Similarly, the lack of clear pooled changes in hip, knee, and ankle angular velocities should not be interpreted as preservation of joint mechanics. These results may reflect the limited and heterogeneous kinematic evidence currently available.

Several biomechanical studies suggest that fatigue-related impairments may emerge earlier in distal segments than in proximal segments. Typical observations include post-fatigue reductions in shank angular velocity with relatively unchanged thigh velocity (Dörge et al., 2002), or early reductions in knee extensor torque and distal speed, followed by later reductions in thigh velocity as fatigue accumulates (Amiri-Khorasani et al., 2011). Fatigue may also reduce knee-extensor peak torque and weaken pre-impact eccentric braking by the hamstrings (Pupo et al., 2014). Together, these changes may reduce the effectiveness of the proximal-to-distal “whip” mechanism and impair ball–foot contact quality (Apriantono et al., 2006).

During simulated match-play protocols, players may maintain foot velocity through temporal reorganization and compensatory adjustments (Greig, 2018). In practice, the thigh upswing lengthens, the shank “whip” phase shortens, and the pelvis opens earlier and more clearly, consistent with an increased proximal drive and altered proximal-to-distal phasing to preserve distal output (Greig, 2018). These findings are consistent with a possible redistribution of timing within the proximal-to-distal sequence, rather than greater joint output. Analyses relying solely on discrete peak values may underestimate fatigue-related changes in timing and coordination (Lees et al., 2010).

Kinematic responses to fatigue are further modulated by both task-related and individual factors, contributing to the heterogeneity observed across studies. At the task level, the source and dose of fatigue are important. Local resistance or isokinetic protocols directly target key determinants of distal speed, particularly the knee extensors and ankle plantarflexors, and are therefore associated with larger declines in foot velocity (Apriantono et al., 2006; Carstensen et al., 2024). In contrast, fatigue induced by countermovement jumps did not significantly reduce foot velocity (Torreblanca-Martinez et al., 2017), suggesting that some fatigue paradigms may not place sufficient demands on the neuromuscular components most closely related to kicking speed. Heat stress magnifies compensation and delays recovery (Vieira et al., 2023). At the individual level, asymmetries between limbs and differences in playing level also shape fatigue responses. The non-dominant foot has been reported to show larger fatigue-related reductions in peak speed and angular velocity, likely reflecting lower baseline shank angular velocity, reduced foot velocity, and less efficient proximal-to-distal energy transfer (Nunome et al., 2006a; Katis et al., 2017). Moreover, higher-level players generally demonstrate superior control of support-leg and pelvic mechanics, as well as greater consistency in ball kicking, characteristics that may help mitigate the effects of fatigue on kicking kinematics (Navandar et al., 2022; Zhang et al., 2025).

### 4.2 Research paradigm

#### 4.2.1 Methodological quality and risk of bias

Overall methodological reporting was moderate but uneven, with several critical procedures lacking standardization and transparency. These shortcomings may weaken internal validity and the stability of pooled effects. “Ball standardization” received the lowest mean score, indicating sparse and unsystematic reporting of ball specifications and pressure, as well as the initial ball state (stationary vs. rolling and its speed). Such variability may plausibly increase measurement error for ball velocity and accuracy and increase between-study heterogeneity. In contrast, “Included players were characterized” received the highest score, suggesting that the sample characteristics were adequately reported.

The risk-of-bias assessment identified “Confounders” as the highest-risk domain. Common confounders that were insufficiently controlled or inadequately reported included factors likely to influence the fatigue intervention (e.g., time of testing, ambient temperature and humidity (Vieira et al., 2023), and the presence or absence of a warm-up (Gelen, 2010)) as well as factors that affect kicking performance (e.g., approach-run length or angle (Kellis et al., 2004), prescribed kicking technique (Brophy et al., 2007), limb dominance (Frontani et al., 2023), footwear (Hennig, 2011), and playing surface (Sánchez-Sánchez et al., 2014)). “Blinding of assessment” was also problematic, particularly in accuracy assessments, where the lack of assessor blinding could have introduced observer bias based on notational analysis (Abbey and Rankin, 2009; Currell et al., 2009).

Substantial uncertainty was observed for “Selective outcome reporting” and “Incomplete outcome data”, reflecting limited preregistration and disclosure of statistical analysis plans, as well as opaque handling of missing data, which limits verifiability and reproducibility. In contrast, “Comparability of the target group” showed low risk in all studies, while “Target group selection” and “Measurement of exposure” were low risk in most studies. These patterns indicate generally clear sample descriptions and fatigue definitions, which support interpretation of the included evidence.

#### 4.2.2 Task design and measurement constraints

Substantial heterogeneity and potential confounding of fatigue effects also stem from differences in kicking task prescription and outcome measurement.

First, task instructions are inconsistent. Only about one quarter of studies specify speed and accuracy requirements (Izquierdo et al., 2020). Most studies emphasize either “maximum speed” (Carstensen et al., 2024) or “hit the target” (Ozimek et al., 2022). Some studies omit instructions entirely (Stone and Oliver, 2009). Because instruction framing can shift the speed–accuracy trade-off, differences in task priority may partly confound fatigue effects with strategic adjustment. Moreover, some studies required players to aim at the entire goal or central zones (Ferraz et al., 2017; Maly et al., 2018), which may underestimate task difficulty and reduce ecological validity, given that goalkeepers typically occupy central areas in match situations. Second, approach conditions and kicking technique were variably controlled. Approach distance and angle influence both ball velocity and accuracy (Kellis et al., 2006). Although the instep kick—typically producing the highest ball velocity—was most frequently examined (Kellis et al., 2006; Kapidžić et al., 2014; Izquierdo et al., 2020), some studies do not standardize kicking technique (Russell et al., 2011; Ozimek et al., 2022; Vieira et al., 2023). Such variability may confound fatigue-related effects: insufficient control increases outcome variance, whereas strict control may constrain habitual movement patterns and reduce ecological validity. Technique selection further influences performance emphasis, with the instep favoring velocity and the inside foot favoring directional control (Levanon and Dapena, 1998). Third, most studies test kicking at short distances, in single scenarios, and under non-opposed conditions. These protocols overlook key contextual constraints such as defensive pressure, visual occlusion, and space–time demands (Forcher et al., 2024). The absence of a goalkeeper or defenders may bias results, and some motor regulatory behaviors may not emerge (Weigelt et al., 2012). In real matches, approach angles and techniques change frequently in response to defender positions, goalkeeper locations, and game context (Van Der Kamp, 2006). The lack of these contextual constraints may partly explain why fatigue effects vary across protocols.

Measurement variability further complicates synthesis. Ball velocity was primarily derived from radar or video-based systems, which show high agreement under specific conditions (Campo et al., 2009), yet few studies reported sampling frequency, measurement error, or device sensitivity (Maly et al., 2018). Foot velocity definitions also varied considerably, particularly regarding marker placement and segment modeling at the foot and ankle (Deschamps et al., 2011; Xu et al., 2015; Malus et al., 2021). Distal kinematic measures are sensitive to modeling choices, and discrepancies in marker schemes can propagate to joint-level outcomes. Additionally, low video sampling frequencies may underestimate peak values around ball impact (Nunome et al., 2006b). Accuracy was assessed using either categorical notational scores (e.g., hit/miss, goal/no goal) or continuous deviation-based metrics. Notational approaches may be less sensitive to subtle changes in placement control than continuous deviation metrics (Russell and Kingsley, 2011; Berjan Bacvarevic et al., 2012), and the same intervention may yield different conclusions depending on the metric used (Russell et al., 2011). Thus, measurement variability may partly explain heterogeneity in the pooled accuracy effect and limit direct comparability.

### 4.3 Limitations

#### 4.3.1 Limitations of the primary evidence

The available evidence is characterized by substantial methodological and conceptual heterogeneity. Fatigue was induced using diverse protocols that varied in duration, intensity, movement pattern, and physiological target. Kicking tasks also differed in instructions, targets, approach conditions, and measurement methods. Consistent with this heterogeneity, the prediction intervals for ball velocity, kicking accuracy, and foot velocity crossed zero, indicating that although the pooled mean effects were negative, future studies conducted under different protocols or task conditions may observe trivial or no fatigue-related impairment. This variability inflates between-study variance, widens prediction intervals, and limits internal validity and the interpretation of pooled effects, particularly when ecological validity is prioritized over experimental control. Participant samples were often small, and because this review was restricted to male players, the findings cannot be generalized to female soccer players. Evidence from clearly defined elite professional cohorts was also limited. Match-related constraints, such as defensive pressure, goalkeeper presence, and time pressure, were rarely included. Many studies also relied on discrete kinematic peak values, which may be insensitive to changes in coordination, support-leg contribution, and proximal-to-distal timing.

#### 4.3.2 Limitations of the present review

Several limitations of the present review should also be acknowledged. The synthesis focused on physical fatigue and excluded mental-fatigue studies. Analyses were not stratified by maturation status, which may obscure developmental differences. Many pre-post correlations were unavailable and were therefore imputed; however, sensitivity analyses using r = 0.30 and r = 0.70 did not change the direction or statistical interpretation of the main pooled effects. To avoid dependent duplicate effects, one effect per outcome was selected from studies with multiple eligible contrasts, but multilevel meta-analysis or robust variance estimation was not applied. Some subgroup analyses were underpowered, and formal moderator tests supported only selected moderators. Finally, the limited number of studies reporting comparable kinematic variables restricted quantitative synthesis, particularly for joint angular velocities, and GRADE certainty ratings were constrained by imprecision and inconsistency.

### 4.4 Practical application

The practical implications should be interpreted with caution because the certainty of evidence was low. The most consistent finding was the reduction in ball velocity, suggesting that coaches should expect shot speed to decrease under high-intensity or accumulated fatigue, although the size of this decrease may vary across players and task settings. When the aim is to improve technique or maximal ball velocity, kicking drills may be better scheduled under low-fatigue conditions. When the aim is to prepare players for the later stages of match play, kicking under accumulated load may be useful. Task instructions should also be considered. Fatigue had a larger effect on ball velocity when players were instructed to kick with maximal speed, whereas accuracy-focused instructions may lead players to reduce execution speed to preserve ball placement. Coaches can use this trade-off to emphasize either maximal output or kicking control under fatigue. Finally, the fatigue protocol should match the training goal: local muscle fatigue protocols are useful when the aim is to examine how lower-limb fatigue affects kicking mechanics, whereas simulated match-demand protocols are more appropriate when the aim is to train kicking after accumulated running load.

## 5 Conclusions

This systematic review and meta-analysis indicate that exercise-induced fatigue is associated with lower ball velocity during soccer kicking and a smaller reduction in kicking accuracy. Evidence for reduced foot velocity is tentative, whereas pooled evidence for changes in hip, knee, and ankle angular velocities remains very uncertain. Formal subgroup analyses indicated that kicking instruction moderated fatigue-related changes in ball velocity and that fatigue protocol moderated changes in kicking accuracy. Other contextual factors, including player level, were not supported by significant between-subgroup tests. Overall, the findings suggest a selective rather than uniform effect of fatigue on kicking performance, but the certainty of evidence remains low across outcomes.

## Data Availability

The datasets analyzed in this study were extracted from previously published articles included in the systematic review and meta-analysis. All original data are available in the cited publications. The extracted meta-analytic datasets are available from the corresponding author upon reasonable request.
