# Supplementary material for: Exercise-induced fatigue and soccer kicking performance: a systematic review and meta-analysis of performance outcomes and contextual moderators
Source: Front Physiol. 2026 Jun 30;17:1818415. doi: 10.3389/fphys.2026.1818415 (PMC13364601; doi:10.3389/fphys.2026.1818415)
Supplement: Supplementary file 1 [file DataSheet1.pdf]

Supplementary Table S1. Summary of studies included in the systematic review

Author	Age	N	Level	Fatigue protocol	Kick type	Target	Instruction	Performance indicators	Fatigue indicators
Abt et al. (1998)	18	6	Amateur	60 minutes of intermittent treadmill exercise with three periods of differing intensity	Trials: 8*4<br>Limb: Dominant<br>Foot: Inside	Distance: 15.6 m<br>entire goal<br>(1.5 m wide)	Shoot at a target goal	→Accuracy	↑Blood lactate<br>↑Heart rate
Ali et al. (2007)	21.3	16	Semi-Pro	90-min LIST with six 15-min blocks of intermittent activity, with 3-min rest periods. (Laboratory)	Trials: 10*3<br>Interval: 60s<br>Ball: Stationary<br>Limb: Both side	Distance: 16m<br>Goal upper corners<br>Area: 1.2*0.8m	Shoot towards the open space of the goal	→Accuracy	↑Heart rate<br>↑RPE<br>↑Blood lactate
Alkhawaldeh, I. M. (2022)	25.1	12	Pro	Cunningham-Faulkner Anaerobic Treadmill Test (8.0 mph, 20% slope until exhaustion)	Trials: 5*2	Distance: 11m<br>divided standard goal (max score 45)	-	↓Approach velocity<br>↓Ball velocity<br>↓Knee's angular velocity<br>↓Accuracy	
Apriantono et al. (2006)	20	7	Univ	Repeated loaded knee extension and flexion motions on a force platform until exhaustion,	Trails: 5*2<br>Ball: Standard<br>Limb: Dominant, Foot: Instep	Distance: 11m<br>Goal center<br>Goal size: 3*2m	Maximal kick toward a goal	↓Ball velocity<br>↓Maximal toe linear velocity<br>↓Peak lower leg angular velocity<br>→Peak thigh angular velocity	
Béliard et al. (2019)	13.5	22	Youth	SAFT90 simulation protocol: 2 halves of 36 min (3 bouts of 12 min each), separated by 15-min half-time (Pitch)	Trails: 2*8	Distance: 10m	Maximal kick toward the radar	↓Ball velocity	↑Heart rate<br>↑RPE<br>↑Blood lactate
Cariolo et al. (2019)	21.1	12	Semi-Pro	Training sessions lasting approximately 124-134 min (Pitch)	Trials: 8*2	Lower/Upper corners	Kick the ball to score	→Accuracy	↑Heart rate
Carstensen et al. (2024)	24.6	16	Pro	5 sets of 10 maximal voluntary concentric and eccentric knee extensions at 60°/s with the kicking leg, 10-second inter-set rest (Laboratory)	Trails: 3*2/10*2<br>Interval: 30s<br>Ball: Standard, Stationery/Rolling<br>Limb: Dominant Foot: Instep	Distance: 11m	1.Maximal force<br>2.Keep accuracy and kick at a minimum of 80% of their measured current maximum speed	↓Ball velocity<br>→Accuracy<br>↓Foot velocity	
De Pablo et al. (2024)	18.2	21	Pro	First 45 minutes of the CST with nine laps at low, medium, and high intensities (Pitch)	Trials: 1*9	Distance: 20m<br>Divided standard goal	Kick to the corner of goal to get higher point	Grouped by time phases:<br>→Goal shooting accuracy<br>A higher intensity:<br>↓Goal shooting accuracy	Grouped by time phases:<br>↑RPE<br>A higher intensity:<br>↑RPE
Draganidis et al. (2013)	20	10	Pro	Low-intensity resistance exercise and high-intensity resistance exercise, 40-45 min duration. (Pitch)	Trails: 3*5*6<br>Ball: Stationery	Distance: 16m<br>Entire goal	Kick into the goal by aiming at different segments	↓Accuracy	
Ferraz et al. (2019)	19.7	24	Semi-Pro	Five 90-s circuits involving jumps, skipping, changes of direction, driving the ball, passing, sprinting, and slow running. 2 min of rest.	Trials: 3*6<br>Ball: Standard<br>Foot: Instep	Distance: 11m<br>Goal center<br>Goal (7.32*2.44m)<br>Area: 1*1m	Maximum force and attempt to hit a target	↓Maximal ball velocity<br>→Accuracy	↑Heart rate<br>↑RPE<br>↑Blood lactate
Ferraz et al. (2012)	27.3	10	Amateur	Five 90-s circuits involving jumps, skipping, changes of direction, passing, sprinting, and slow running. followed by 90 seconds of rest before the start of the next 90 s circuit.	Trials: 3*6<br>Ball: Standard<br>Foot: Instep	Distance: 7m<br>Goal center<br>Goal (3*2m)<br>Area: 1*1m	Maximum force and attempt to hit a target	↓Maximal ball velocity<br>↓Average ball velocity	↑Heart rate<br>↑RPE<br>↑Blood lactate
Ferraz et al. (2017)	19.7	12	Semi-pro	Five circuits at varying intensities (slowest, a bit slower, preferred tempo, a bit faster, fastest) involving jumping, skipping, changes of direction, passing, sprinting, and slow running. 5 min rest between each circuit.	Trials: 3*7<br>Ball: Standard<br>Foot: Instep	Distance: 11m<br>Goal center<br>Goal (7.32*2.44m)<br>Area: 1*1m	Maximum force and attempt to hit a target	Only at the highest intensity<br>↓Average ball velocity<br>All intensities<br>→Accuracy	↑Heart rate<br>↑RPE<br>↑Blood lactate
Gaspar et al. (2019)	13.8	20	Elite Youth	35-min simulated 11-a-side football match (Pitch)	Trails: 3*2/12*2<br>Interval: 60s<br>Ball: Stationary<br>Limb: Dominant<br>Approach: 5m	Distance: 11m<br>1.Entire goal<br>2.Upper corners<br>(0.7*0.7m)	1.Maximal effort kick<br>2. Kick into a goal to achieve as high a score as possible	→Ball velocity<br>→Accuracy	
Gharbi et al. (2017)	14.6	10	Youth	Repeated-dribbling sprint test: 10 × 20 m maximal slalom sprints with 20 s of recovery. (Pitch)	Trails: 10*2	Distance: 6.1m<br>Entire goal	-	→Accuracy	↑RPE
Gopalakrishnan et al. (2024)	19.2	30	Pro	90-min TSAFT90, a football-specific simulation (Indoor)	Trial: 1*12<br>Ball: Rolling	Distance: 11m	-	↓Ball velocity	↑RPE<br>↑Blood lactate
Greig (2018)	20.8	10	Pro	90-min intermittent treadmill protocol based on soccer match-play velocity profile, repeated six times with a 15-min halftime interval	Trials: 1*8<br>Ball: Stationary<br>Approach: Self-selected	-	Maximal velocity kick	→Kicking foot velocity	
Izquierdo et al. (2020)	17.6	198	Elite Youth	Official federation matches<br>(Pitch)	Trials: 3*3<br>Interval: 60s<br>Foot: Instep<br>Approach: 2 steps	Distance: 5m	Maximum ball speed when aiming at the goal	↓Ball velocity	

Author	Age	N	Level	Fatigue protocol	Kick type	Target	Instruction	Performance indicators	Fatigue indicators
Juárez et al. (2011)	16.1	21	Elite Youth	20-min treadmill run at 80% of individual maximum heart rate (Laboratory)	Trials: 3<br>Interval: 30s<br>Limb: Dominant<br>Foot: Instep<br>Approach: 4-5m	Distance: 5m	Maximal soccer kick	→Maximum ball velocity<br>→Maximum linear velocity of the toe<br>→Maximum linear velocity of the ankle<br>→Maximum linear velocity of the knee<br>→Maximum linear velocity of the hip<br>→Linear velocity of the toe at ball contact	
Katis et al. (2014)	26.3	10	Amateur	Treadmill running till exhaustion with increasing speed and incline (Laboratory)	Trials: 3*2,<br>Interval: 30s<br>Ball: Stationary<br>Foot: Instep<br>Approach: 1 step, 45 degree	Distance: 7m<br>Goal center	As fast and hard as possible aiming at the center of the goalpost.	Significantly declined in first two post-fatigue trials<br>↓Ball velocity<br>↓Maximal ankle, knee and hip linear velocities<br>↓Ankle and knee angular displacement	
Katis et al. (2017)	24.5	10	Amateur	Treadmill running till exhaustion with increasing speed and incline (Laboratory)	Trials: 2*2<br>Interval: 15s<br>Ball: Stationary, Standard<br>Limb: Both sides<br>Foot: Instep<br>Approach: 1 step, 45 degree	Distance: 7m<br>Goal center	As fast and hard as possible aiming at the center of the goalpost.	↓Ball velocity<br>↓Ankle, knee and hip linear velocities<br>↓Ankle, knee and hip angle velocities	
Kellis et al. (2006)	22.6	10	Amateur	90 min intermittent exercise protocol with two 45 min periods separated by 15 min rest, including walking, sprinting, jogging, and running	Trials: 3*3<br>Interval: 30s<br>Foot: Instep<br>Approach: 2 steps	Distance: 11m<br>Entire goal<br>(2.5*7.5m)	As powerful as possible, towards a goal area.	↓Ball velocity<br>↓Foot velocity<br>↓Maximum Angular Velocity of the Shank<br>→Maximum Angular Velocity of the Thigh	↑Heart rate<br>↑Blood lactate
Maly et al. (2018)	22.4	20	Elite	Yo-Yo IR1 (Pitch)	Trials: 3*2<br>Ball: Stationary, Standard<br>Limb: Dominant<br>Foot: Instep	Distance: 11m<br>Entire goal	Maximum effort kick to the centre of the goal	↓Ball velocity<br>→Kicking accuracy	
McMorris et al. (2000)	20	12	Univ	Incremental cycle ergometer test to exhaustion	Trials: 3*3<br>Ball: Stationary<br>Approach: 1m	Distance: 7m<br>Height:0.24m<br>Width: 3.3m	Aim for the center of the target.	→Accuracy	↑Heart rate
Mor et al. (2022)	19.5	8	Amateur	Running Anaerobic Sprint Test: six consecutive 35-meter sprints with 10-second breaks between each sprint.	Trials: 2*2<br>Ball: Stationary<br>Limb: Dominant<br>Foot: Instep	Distance: 20 m<br>Entire goal<br>(7.32*2.44m)	Kick as fast as possible aiming at the goal	↓Ball velocity	↑Heart rate
Owen et al. (2013)	22.2	13	Semi-Pro	LIST: six 15-minute periods of intermittent activity interspersed with five 3-minute recovery periods<br>(Indoor)	Trials: 5(left and right foot) *2,<br>Interval: 1min<br>Ball: Rolling<br>Limb: Both sides	Distance: 16.5m<br>Upper corners of entire goal<br>Area: 1.2*0.8m	Shoot the ball at targets in a full-sized goal	↓Ball velocity<br>→Accuracy	↑Heart rate<br>↑RPE
Ozimek et al. (2022)	14.5	24	Youth	5 sets of back squats at 50% body weight, 40 seconds per set, 56 bpm metronome tempo, 10-12 repetitions per set; pROM limited knee flexion to 100-110 degrees, fROM full range<br>(Pitch)	Trails: 5*5*2<br>Interval: 4min	Distance: 11m<br>Area: 1*1m	Kick to hit target	↓Accuracy	↑Blood lactate
Radman et al. (2016)	22.9	28	Semi-Pro	Progressive discontinuous shuttle-run starting at 8 km/h, increasing by 1 km/h every 3 min until exhaustion, with 3 min breaks and kicking trials post each increment. (Pitch)	Trials: 10*7<br>Interval: 6s<br>Goal size: 3*2<br>Ball: Stationary, Standard<br>Limb: Dominant<br>Approach: 2 steps	Distance: 16.5m<br>Score of entire goal<br>(7.32*2.44m)<br>Area: 48.8*48.8 cm	Accurately hit the most distant scoring zones of the goal while keeping kicking at the velocity	Above second lactate threshold (LT2)<br>↓Kicking accuracy<br>↓Ball velocity<br>↓Kicking quality<br>No significant change below LT2	↑Heart rate<br>↑RPE<br>↑Blood lactate
Russell et al. (2011)	18.1	15	Elite Academy	90-minute SMS with two 45-min halves separated by 15-min half-time, including intermittent high-intensity movements.<br>(Indoor)	Trials: 4*8<br>Interval: 30s<br>Ball: Rolling, Standard	Distance: 15m<br>Lower/Upper corners of entire goal (7.33*2.44m)<br>Area: 1*0.5m	Kick the ball toward one of targets	↓Ball velocity<br>↓Kicking accuracy	↑Blood lactate
Russell et al. (2012)	18	15	Elite Academy	90-minute SMS with two 45-min halves separated by 15-min half-time, including intermittent high-intensity movements.<br>(Indoor)	Trials: 4*4<br>Ball: Rolling, Standard	Distance: 15m<br>Lower/Upper corners of entire goal (7.33*2.44m)<br>Area: 1*0.5m	Kick the ball toward one of targets	↓Ball velocity<br>→Kicking accuracy	↑Heart rate<br>↑RPE<br>↑Blood lactate
Sánchez-Sánchez et al. (2014)	22.4	18	Amateur	Repeated Sprint Ability test.<br>(Pitch)	Trails: 2*2<br>Interval: 60s<br>Ball: Stationary, Standard	Distance: 11m	The fastest speed possible	↓Ball velocity	↑Blood lactate
Stevenson et al. (2017)	20	22	Univ	120-min SMS with two 45-min halves and two 15-min extra periods. (Indoor)	Trials: 2*7<br>Ball: Rolling<br>Limb: Dominant	Distance: 11m<br>Lower/Upper corners of entire goal (7.33*2.44m)	Kick toward one of four random targets	↓Ball velocity<br>→Accuracy	↑Heart rate<br>↑RPE<br>↑Blood lactate

Author	Age	N	Level	Fatigue protocol	Kick type	Target	Instruction	Performance indicators	Fatigue indicators
Stone and Oliver (2009)	20.7	9	Semi-Pro	Three 15-min bouts of a modified LIST with 3-min rest periods. (Pitch)	Trials: 10 (5 left, 5 right) * 2<br>Interval: 30s<br>Ball: Rolling<br>Limb: Both sides	Distance: 16.5m<br>Upper corners of entire goal (2.44*7.32m)<br>Area: 1.2*0.8m	-	↓Kicking accuracy	↑Heart rate
Torreblanca-Martínez et al. (2017)	17.5	10	Elite Youth	Continuous and maximum CMJ were carried out on the dynamometric platform for 15 second	Trial: 1*3<br>Foot: Instep	-	Maximal kick	→Foot velocity	
Vieira et al. (2023)	16.3	15	Elite Youth	Repeated high intensity running protocol with 10 × 30 m all-out sprints, 30 s intervals, 25 s low-intensity return to start line. (Pitch)	Trials: 3*2<br>Interval: 40s<br>Ball: Stationery, Standard<br>Approach: 3.5m, 45-degree	Distance: 18m<br>Area: 1*1m	Maximal velocity, aimed at the center of target.	↓Average ball velocity<br>↓Peak ball velocity<br>↓Accuracy<br>→Foot velocity<br>→Non-preferred hip velocity<br>→Peak knee angular velocity	↑RPE
Yasar et al. (2025)	21.7	21	Amateur	Yo-Yo IR1	Trial: 3*6<br>Ball: Stationary	Distance: 10m<br>Center of the goal (2*3m)<br>Area:0.5*0.5m	Shoot at the center of the prepared target	↓Accuracy	↑Heart rate

Note. ↓ drop, ↑ increase (both with statistical significance); → maintenance (without statistical significance); RPE – rate of perception of effort; Pro – Professional; Univ – University; CMJ – Counter Movement Jump; LIST – Loughborough Intermittent Shuttle Test; SAFT90 / TSAFT90 – Soccer-Specific Aerobic Field Test (90 min); Yo-Yo IR1 – Yo-Yo Intermittent Recovery Test Level 1; LT2 – second lactate threshold; pROM – partial range of motion; fROM – full range of motion; CST – Copenhagen Soccer Test; SMS – Soccer Match Simulation.

Supplementary Table S2. Studies Search was conducted in April 2025

Database	Search query	Result
**PubMed**	(<br>(soccer[Title/Abstract] OR football*[Title/Abstract] OR "association football"[Title/Abstract])<br>AND<br>(fatigu*[Title/Abstract] OR exercis*[Title/Abstract] OR exhausti*[Title/Abstract] OR "match demands"[Title/Abstract] OR "post-match"[Title/Abstract] OR "match-related fatigue"[Title/Abstract])<br>AND<br>(kick*[Title/Abstract] OR shoot*[Title/Abstract] OR skill*[Title/Abstract] OR technical[Title/Abstract])<br>AND<br>(biomechanic*[Title/Abstract] OR kinematic*[Title/Abstract] OR "motion analysis"[Title/Abstract] OR "3D motion"[Title/Abstract] OR velocity[Title/Abstract] OR speed[Title/Abstract] OR accuracy[Title/Abstract] OR precision[Title/Abstract] OR performance[Title/Abstract])<br>)	324
**Scopus**	TITLE-ABS-KEY((soccer OR football* OR "association football")<br>AND<br>(fatigu* OR exercis* OR exhausti* OR "match demands" OR "post-match" OR "match-related fatigue")<br>AND<br>(kick* OR shoot* OR skill* OR technical)<br>AND<br>(biomechanic* OR kinematic* OR "motion analysis" OR "3D motion" OR velocity OR speed OR accuracy OR precision OR performance))	1142
**SPORTDiscus**	AB (soccer OR football* OR "association football")<br>AND<br>AB (fatigu* OR exercis* OR exhausti* OR "match demands" OR "post-match" OR "match-related fatigue")<br>AND<br>AB (kick* OR shoot* OR skill* OR technical)<br>AND<br>AB (biomechanic* OR kinematic* OR "motion analysis" OR "3D motion" OR velocity OR speed OR accuracy OR precision OR performance)	449
**Web of Science**	TS = ((soccer OR football* OR "association football")<br>AND<br>(fatigu* OR exercis* OR exhausti* OR "match demands" OR "post-match" OR "match-related fatigue")<br>AND<br>(kick* OR shoot* OR skill* OR technical)<br>AND<br>(biomechanic* OR kinematic* OR "motion analysis" OR "3D motion" OR velocity OR speed OR accuracy OR precision OR performance))	1135

Supplementary Table S3. Studies Search was conducted in December 2025

Database	Search query	Result
**PubMed**	(<br>(soccer[Title/Abstract] OR football*[Title/Abstract] OR "association football"[Title/Abstract])<br>AND<br>(fatigu*[Title/Abstract] OR exercis*[Title/Abstract] OR exhausti*[Title/Abstract] OR "match demands"[Title/Abstract] OR "post-match"[Title/Abstract] OR "match-related fatigue"[Title/Abstract])<br>AND<br>(kick*[Title/Abstract] OR shoot*[Title/Abstract] OR skill*[Title/Abstract] OR technical[Title/Abstract])<br>AND<br>(biomechanic*[Title/Abstract] OR kinematic*[Title/Abstract] OR "motion analysis"[Title/Abstract] OR "3D motion"[Title/Abstract] OR velocity[Title/Abstract] OR speed[Title/Abstract] OR accuracy[Title/Abstract] OR precision[Title/Abstract] OR performance[Title/Abstract])<br>)	53
**Scopus**	TITLE-ABS-KEY((soccer OR football* OR "association football")<br>AND<br>(fatigu* OR exercis* OR exhausti* OR "match demands" OR "post-match" OR "match-related fatigue")<br>AND<br>(kick* OR shoot* OR skill* OR technical)<br>AND<br>(biomechanic* OR kinematic* OR "motion analysis" OR "3D motion" OR velocity OR speed OR accuracy OR precision OR performance))	128
**SPORTDiscus**	AB (soccer OR football* OR "association football")<br>AND<br>AB (fatigu* OR exercis* OR exhausti* OR "match demands" OR "post-match" OR "match-related fatigue")<br>AND<br>AB (kick* OR shoot* OR skill* OR technical)<br>AND<br>AB (biomechanic* OR kinematic* OR "motion analysis" OR "3D motion" OR velocity OR speed OR accuracy OR precision OR performance)	34
**Web of Science**	TS = ((soccer OR football* OR "association football")<br>AND<br>(fatigu* OR exercis* OR exhausti* OR "match demands" OR "post-match" OR "match-related fatigue")<br>AND<br>(kick* OR shoot* OR skill* OR technical)<br>AND<br>(biomechanic* OR kinematic* OR "motion analysis" OR "3D motion" OR velocity OR speed OR accuracy OR precision OR performance))	118

Supplementary Table S4. Methodological Quality of Included Studies

Study	Q1	Q2	Q3	Q4	Q5	Q6	Q7	Q8	Q9	Total	Quality score (%)
Abt et al. (1998)	1	2	1	1	0	2	2	1	1	11	61%
Ali et al. (2007)	1	2	1	1	1	1	2	2	2	13	72%
Alkhawaldeh, I. M. (2022)	2	2	1	1	0	1	1	1	1	10	56%
Apriantono et al. (2006)	1	2	2	1	2	1	1	2	1	13	72%
Béliard et al. (2019)	1	2	1	1	0	2	2	1	2	14	78%
Cariolo et al. (2019)	1	2	1	0	1	1	2	1	2	11	61%
Carstensen et al. (2024)	2	2	2	2	1	2	2	2	2	17	94%
De Pablo et al. (2024)	1	2	1	1	1	1	2	2	2	13	72%
Draganidis et al. (2013)	2	2	0	1	1	1	2	2	1	12	67%
Ferraz et al. (2019)	2	2	1	1	2	2	2	2	2	16	89%
Ferraz et al. (2012)	2	2	1	1	2	2	2	2	2	16	89%
Ferraz et al. (2017)	2	2	1	1	2	2	2	2	2	16	89%
Gaspar et al. (2019)	1	2	2	1	1	2	2	1	2	14	78%
Gharbi et al. (2017)	1	2	1	0	1	2	2	1	1	11	61%
Gopalakrishnan et al. (2024)	1	2	1	1	0	1	2	1	2	11	61%
Greig (2018)	2	2	1	1	2	1	2	2	2	15	83%
Izquierdo et al. (2020)	2	2	1	1	1	2	2	2	2	15	83%
Juárez et al. (2011)	1	2	1	1	2	1	2	1	2	13	72%
Katis et al. (2014)	2	2	1	1	2	2	2	1	2	15	83%
Katis et al. (2017)	2	2	1	2	2	2	2	1	2	16	89%
Kellis et al. (2006)	2	2	1	0	2	2	2	1	2	14	78%
Maly et al. (2018)	1	2	1	1	2	2	2	2	2	15	83%
McMorris et al. (2000)	1	2	1	0	1	2	2	1	2	12	67%
Mor et al. (2022)	1	2	1	1	1	2	2	1	2	13	72%
Owen et al. (2013)	2	2	2	0	1	2	2	2	2	15	83%
Ozimek et al. (2022)	2	2	2	0	2	2	2	1	2	15	83%
Radman et al. (2016)	2	2	2	1	2	2	2	1	2	16	89%
Russell et al. (2011)	2	2	1	0	2	2	2	1	2	14	78%
Russell et al. (2012)	1	2	1	1	2	2	2	1	2	14	78%
Sánchez-Sánchez et al. (2014)	1	2	1	2	1	2	2	1	2	14	78%
Stevenson et al. (2017)	2	2	1	1	1	2	2	2	2	15	83%
Stone and Oliver (2009)	1	2	2	1	1	1	2	2	2	14	78%
Torreblanca-Martínez et al. (2017)	2	2	0	0	2	2	2	2	1	13	72%
Vieira et al. (2023)	2	2	2	2	1	1	2	2	2	16	89%
Yaşar et al. (2025)	2	2	1	1	0	2	2	2	2	14	78%

Q1 Study objective(s) is/are clearly set out. Q2 Included players were characterized (sample size, age, competitive level). Q3 Detailed design regarding kicking task (objective instruction, lower limb used, approach run, target specification, trials and whether defenders participated). Q4 Ball standardization [sizes, inflation pressure, condition (e.g. rolling/stationary)] and location where data collection took place. Q5 Validity/reliability or error of measurement system/equipment is not stated, mentioned as a citation of previous study(s) or measured under local conditions. Q6 Dependent variables defined. Q7 Statistical treatment to analyze main outcomes were deemed appropriate. Q8 Data are detailed (mean and standard deviation, percent change/difference, effect size/mechanistic magnitude-based inference). Q9 Conclusions are insightful (clear, practical applications, and future directions).

Criteria used in methodological quality assessments: Yes = 2; Partially = 1; No = 0. Strict rules applied to Q2 (No information = 0 point; 1 – 2 items described = 1 point; all items described = 2 points); Q3 (0 – 2 items described = 0 point; 3 – 4 items described = 1 point; 5 – 6 items described = 2 points); Q4 (No information = 0 point; 1 – 2 items described = 1 point; 3 – 4 items described = 2 points); and Q8 [description of mean, standard deviation and null hypothesis significance test (p-value) = 1 point; also included effect size/magnitude-based inferences = 2 points].

Supplementary Table S5. Handling of pre-post correlations and dependent effect sizes

Study	Outcome	r source	r used	Sensitivity r	Multiple-effect decision
Abt et al. (1998)	Accuracy	Not reported/not derivable; imputed	0.50	0.30, 0.70	Mixed/control diet selected; high-CHO condition excluded to avoid dependent duplicate effects.
Apriantono et al. (2006)	Ball velocity<br>Foot velocity	Not reported/not derivable; imputed	0.50	0.30, 0.70	No selection among multiple effects was required; one eligible pre–post contrast was available for ball velocity.
Cariolo et al. (2019)	Accuracy	Not reported/not derivable; imputed	0.50	0.30, 0.70	Ad libitum hydration and auditive-signal task selected; recommended hydration and visual-decision task excluded to avoid dependent duplicate effects.
Carstensen et al. (2024)	Ball velocity<br>Accuracy<br>Foot velocity	Derived from 95% CI of paired change score	0.744<br>-0.177<br>0.854	Not varied<br>Not varied<br>Not varied	Intervention/fatigue condition selected; control condition excluded from pre-post fatigue synthesis.
De Pablo et al. (2024)	Accuracy	Not reported/not derivable; imputed	0.50	0.30, 0.70	Phase 1 vs Phase 3 selected for time-based fatigue comparison; intensity-based comparison excluded from main pre-post synthesis to avoid dependent duplicate effects.
Draganidis et al. (2013)	Accuracy	Not reported/not derivable; imputed	0.50	0.30, 0.70	High-intensity resistance exercise, immediate post-exercise selected; low-intensity/control and later recovery time points excluded to avoid dependent duplicate effects.
Ferraz et al. (2019)	Ball velocity<br>Accuracy	Not reported/not derivable; imputed	0.50	0.30, 0.70	Without-knowledge condition selected; with-knowledge condition excluded to avoid condition-related duplicate effects. Pre-fatigue vs after circuit-1 fatigue stage selected to represent acute fatigue condition.
Ferraz et al. (2012)	Ball velocity	Not reported/not derivable; imputed	0.50	0.30, 0.70	Pre-fatigue vs after circuit-1 fatigue stage selected to represent acute fatigue condition.
Ferraz et al. (2017)	Ball velocity<br>Accuracy	Not reported/not derivable; imputed	0.50	0.30, 0.70	After warm-up vs fastest-intensity condition selected because this study compared randomized exercise intensities rather than cumulative fatigue stages; lower-intensity conditions excluded to avoid dependent duplicate effects.
Gharbi et al. (2017)	Accuracy	Not reported/not derivable; imputed	0.50	0.30, 0.70	Passive-recovery condition selected; active-recovery condition excluded to avoid dependent duplicate effects.
Gopalakrishnan et al. (2024)	Ball velocity	Not reported/not derivable; imputed	0.50	0.30, 0.70	Pre-TSAFT90 vs immediately post-TSAFT90 selected; 24-h follow-up used only for recovery biomarkers, not ball velocity.
Greig (2018)	Foot velocity	Not reported/not derivable; imputed	0.50	0.30, 0.70	Pre-exercise vs end-protocol value selected; intermediate 15-min time points excluded to avoid dependent duplicate effects.
Izquierdo et al. (2020)	Ball velocity	Not reported/not derivable; imputed	0.50	0.30, 0.70	T1 pre-match vs T3 post-match selected; T2 half-time and position-specific subgroup effects excluded to avoid dependent duplicate effects.
Juárez et al. (2011)	Ball velocity<br>Foot velocity	Not reported/not derivable; imputed	0.50	0.30, 0.70	One eligible pre–post contrast was available for ball velocity, Toe velocity at ball contact was selected as the foot-velocity outcome.
Katis et al. (2014)	Ball velocity	Not reported/not derivable; imputed	0.50	0.30, 0.70	First pre-fatigue trial vs first post-fatigue trial selected to capture the immediate fatigue effect; later post-fatigue trials excluded because they include short-term recovery.
Katis et al. (2017)	Ball velocity<br>Hip angular velocity<br>Knee angular velocity<br>Ankle angular velocity	Not reported/not derivable; imputed	0.50	0.30, 0.70	Preferred-leg pre-fatigue vs post-fatigue selected; non-preferred leg excluded to avoid dependent duplicate effects.
Kellis et al. (2006)	Ball velocity<br>Foot velocity<br>Hip angular velocity<br>Knee angular velocity<br>Ankle angular velocity	Not reported/not derivable; imputed	0.50	0.30, 0.70	Pre-exercise vs post-protocol selected; middle-protocol value excluded to avoid dependent duplicate effects.
Maly et al. (2018)	Ball velocity<br>Accuracy	Derived from paired t value	0.190<br>0.181	Not varied<br>Not varied	Preferred-leg PRE vs preferred-leg POST selected.
McMorris et al. (2000)	Accuracy	Not reported/not derivable; imputed	0.50	0.30, 0.70	Rest vs maximum power output selected; epinephrine-threshold condition excluded to avoid dependent duplicate effects.
Mor et al. (2022)	Ball velocity	Not reported/not derivable; imputed	0.50	0.30, 0.70	PLA pre-supplementation Rest vs Exhausted selected; BCAA/creatine groups and post-supplementation comparisons excluded to avoid supplement confounding and dependent duplicate effects.
Owen et al. (2013)	Ball velocity<br>Accuracy	Not reported/not derivable; imputed	0.50	0.30, 0.70	Ad libitum-fluid trial selected; no-fluid and prescribed-fluid trials excluded to avoid dependent duplicate effects.
Ozimek et al. (2022)	Accuracy	Not reported/not derivable; imputed	0.50	0.30, 0.70	pROM pre–post contrast was selected, fROM was excluded to avoid multiple dependent effects from the same study

Study	Outcome	r source	r used	Sensitivity r	Multiple-effect decision
Radman et al. (2016)	Ball velocity<br>Accuracy	Not reported/not derivable; imputed	0.50	0.30, 0.70	Experimental baseline vs zone 5 selected; control condition and lower-intensity zones excluded to avoid dependent duplicate effects.
Russell et al. (2011)	Ball velocity<br>Accuracy	Not reported/not derivable for selected endpoint contrast; imputed	0.50	0.30, 0.70	Pre 1st half vs Post 2nd half endpoint selected; first-half vs second-half comparison and intermediate time points excluded to avoid dependent duplicate effects.
Russell et al. (2012)	Ball velocity<br>Accuracy	Not reported/not derivable; imputed	0.50	0.30, 0.70	Placebo trial, initial vs final shooting assessment selected; CHO trial excluded to avoid carbohydrate-supplementation confounding; intermediate time points excluded to avoid dependent duplicate effects.
Sánchez-Sánchez et al. (2014)	Ball velocity	Not reported/not derivable; imputed	0.50	0.30, 0.70	Overall pre-RSA vs post-RSA mean across the four artificial turf systems selected; surface-specific effects not entered separately to avoid dependent duplicate effects from the same players.
Stevenson et al. (2017)	Ball velocity<br>Accuracy	Not reported/not derivable; imputed	0.50	0.30, 0.70	Placebo trial, Pre 1st half vs Post 2nd half selected; PSE/MDX trials and extra-time value excluded to avoid supplementation confounding and dependent duplicate effects.
Stone & Oliver (2009)	Accuracy	Derived from reported SD of change for no-time-limit score	0.58	Not varied	No-time-limit score selected to isolate shooting accuracy/score from the movement-time constraint; with-time-limit score excluded to avoid duplicate effects.
Torreblanca-Martinez et al. (2017)	Foot velocity	Directly calculated from individual participant data in Table 1 / consistent with reported SD of change	0.76	Not varied	Foot velocity at impact without fatigue vs with fatigue selected; no duplicate kicking outcomes.
Vieira et al. (2023)	Ball velocity	Derived from reported paired comparison	0.76	Not varied	Experiment 1 Pre-RHIR vs Post-RHIR selected;
	Accuracy		0.32	Not varied	
	Foot velocity	Not reported/not derivable; imputed	0.50	0.30, 0.70	
	Peak knee angular velocity				
Yaşar et al. (2025)	Accuracy	Not reported/not derivable; imputed	0.50	0.30, 0.70	Rest vs Level-5 selected to represent the highest workload/HR condition; intermediate Yo-Yo IR1 levels excluded to avoid dependent duplicate effects.

Note. For within-participant pre-post designs, the pre-post correlation coefficient (r) was extracted when reported or derived when sufficient paired statistics were available. When the SD of the change score was reported, r was calculated as: $r = (SDpre^2 + SDpost^2 - SDchange^2) / (2 \times SDpre \times SDpost)$. When confidence intervals for paired mean changes were reported, the SD of change was first derived from the standard error of the change score. If r could not be reliably derived, an imputed value of 0.50 was used in the main analysis, with sensitivity analyses using r = 0.30 and r = 0.70. When multiple eligible effects were available within the same study, one effect was selected to avoid dependent duplicate effects.

Abbreviations: CHO, carbohydrate; CMA, Comprehensive Meta-Analysis; COOL, cooling condition; LSST, Loughborough Soccer Shooting Test; PL/PLA, placebo; RHIR, repeated high-intensity running; RSA, repeated sprint ability; SEM, standard error of the mean; SD, standard deviation.

Supplementary Table S6. Sensitivity analyses using alternative imputed pre-post correlations

Outcome	Imputed pre-post r	k	Model	Hedges' g	SE	95% CI	p value	I² (%)	Tau²	Change in inference
Ball velocity	0.30	20	Random effects	-0.573	0.101	-0.771 to -0.375	<0.001	62.1	0.107	No
Ball velocity	0.50	20	Random effects	-0.588	0.102	-0.788 to -0.389	<0.001	72.2	0.130	Reference
Ball velocity	0.70	20	Random effects	-0.643	0.107	-0.852 to -0.434	<0.001	82.5	0.167	No
Kicking accuracy	0.30	19	Random effects	-0.273	0.096	-0.461 to -0.086	0.004	46.1	0.078	No
Kicking accuracy	0.50	19	Random effects	-0.266	0.094	-0.450 to -0.082	0.005	58.3	0.094	Reference
Kicking accuracy	0.70	19	Random effects	-0.254	0.091	-0.431 to -0.076	0.005	72.0	0.108	No
Foot velocity	0.30	7	Random effects	-0.335	0.139	-0.606 to -0.063	0.016	37.2	0.046	No
Foot velocity	0.50	7	Random effects	-0.348	0.138	-0.618 to -0.078	0.012	56.7	0.068	Reference
Foot velocity	0.70	7	Random effects	-0.432	0.163	-0.751 to -0.113	0.008	73.5	0.128	No

Note. The imputed pre-post correlation was varied only for studies in which the correlation could not be extracted or derived. Study-specific extracted or derived correlations were retained unchanged. The r = 0.50 model was used as the primary analysis. All models are random-effects models. Negative Hedges' g values indicate poorer post-fatigue performance relative to pre-fatigue performance. Change in inference refers to whether the direction and statistical interpretation of the pooled effect changed compared with the primary r = 0.50 analysis.

Supplementary Table S7. Study-level coding of subgroup categories

Study	Outcome(s) entered in meta-analysis	Fatigue protocol category	Player level	Ball-velocity measure	Kicking instruction	Accuracy metric type	Included in subgroup analyses
Abt et al. (1998)	ACC	SMD	Amateur	NA	Accuracy	Notational	Yes
Ali et al. (2007)	Systematic review only	SMD	Semi-Pro	NA	Accuracy	Notational	No: not meta-analysed
Alkhawaldeh, I. M. (2022)	Systematic review only	GE	Pro	NR	Not reported	Notational	No: not meta-analysed
Apriantono et al. (2006)	BV; Foot LV	LMF	Univ	Video/motion	BVmax	NA	Yes
Béliard et al. (2019)	Systematic review only	SMD	Youth	Radar gun	BVmax	NA	No: not meta-analysed
Cariolo et al. (2019)	ACC	SMD	Semi-Pro	NA	Accuracy	Notational	Yes
Carstensen et al. (2024)	BV; ACC; Foot LV	LMF	Pro	Radar gun	BVA	Deviation	Yes
De Pablo et al. (2024)	ACC	SMD	Pro	NA	Accuracy	Notational	Yes
Draganidis et al. (2013)	ACC	LMF	Pro	NA	Accuracy	Notational	Yes
Ferraz et al. (2019)	BV; ACC	SSE	Semi-Pro	Radar gun	BVA	Deviation	Yes
Ferraz et al. (2012)	BV	SSE	Amateur	Radar gun	BVA	NA	Yes
Ferraz et al. (2017)	BV; ACC	SSE	Semi-Pro	Radar gun	BVA	Deviation	Yes
Gaspar et al. (2019)	Systematic review only	SMD	Elite Youth	NR	Multiple tasks	Notational	No: not meta-analysed
Gharbi et al. (2017)	ACC	SSE	Youth	NA	Not reported	Deviation	Partial: instruction not reported
Gopalakrishnan et al. (2024)	BV	SMD	Pro	Radar gun	Not reported	NA	Partial: instruction not reported
Greig (2018)	Foot LV	SMD	Pro	NA	BVmax	NA	NA: kinematics only
Izquierdo et al. (2020)	BV	SMD	Pro	Radar gun	BVmax	NA	Yes
Juárez et al. (2011)	BV; Foot LV	Unclassified	Elite Youth	Video/motion	BVmax	NA	Partial: protocol unclassified
Katis et al. (2014)	BV	GE	Amateur	Video/motion	BVmax	NA	Yes
Katis et al. (2017)	BV; Hip AV; Knee AV; Ankle AV	GE	Amateur	Video/motion	BVmax	NA	Yes
Kellis et al. (2006)	BV; Foot LV; Hip AV; Knee AV; Ankle AV	SMD	Amateur	Video/motion	BVmax	NA	Yes
Maly et al. (2018)	BV; ACC	GE	Elite	Radar gun	BVmax	Deviation	Yes
McMorris et al. (2000)	ACC	GE	Univ	NA	Accuracy	Deviation	Yes
Mor et al. (2022)	BV	SSE	Amateur	Radar gun	BVmax	NA	Yes
Owen et al. (2013)	BV; ACC	SMD	Semi-Pro	Radar gun	BVA	Notational	Yes
Ozimek et al. (2022)	ACC	LMF	Youth	NA	Accuracy	Notational	Yes
Radman et al. (2016)	BV; ACC	GE	Semi-Pro	Radar gun	BVA	Notational	Yes
Russell et al. (2011)	BV; ACC	SMD	Elite Academy	Video/motion	BVA	Deviation	Yes
Russell et al. (2012)	BV; ACC	SMD	Elite Academy	Video/motion	BVA	Deviation	Yes
Sánchez-Sánchez et al. (2014)	BV	SSE	Amateur	Radar gun	BVmax	NA	Yes
Stevenson et al. (2017)	BV; ACC	SMD	Univ	Video/motion	BVA	Deviation	Yes
Stone and Oliver (2009)	ACC	SMD	Semi-Pro	NA	Not reported	Notational	Partial: instruction not reported
Torreblanca-Martínez et al. (2017)	Foot LV	LMF	Elite Youth	NA	BVmax	NA	NA: kinematics only
Vieira et al. (2023)	BV; ACC; Foot LV; Knee AV	SSE	Elite Youth	Video/motion	BVA	Deviation	Yes
Yaşar et al. (2025)	ACC	GE	Amateur	NA	Accuracy	Deviation	Yes

Note. ACC, kicking accuracy; AV, angular velocity; BV, ball velocity; Foot LV, foot linear velocity; GE, graded exhaustion; LMF, local muscle fatigue; NA, not applicable; NR, not reported; SMD, simulated match demand; SSE, soccer-specific exercise; BVA, combined ball-velocity and accuracy instruction; BVmax, maximal ball-velocity instruction. Only studies that could be classified were included in each subgroup analysis.

Supplementary Table S8. Summary of findings and certainty of evidence for the effects of fatigue on soccer kicking outcomes

Outcome	k	Participants	Hedges' g (95% CI)	95% PI	I^2 (%)	GRADE certainty	Reasons for downgrading
Ball velocity	20	516	-0.589 (-0.789, -0.388)	(-1.38, 0.20)	72.2	Low	Risk of bias; substantial heterogeneity.
Kicking accuracy	19	303	-0.265 (-0.449, -0.081)	(-0.94, 0.41)	58.2	Low	Risk of bias; moderate heterogeneity.
Foot velocity	7	89	-0.348 (-0.618, -0.078)	(-1.11, 0.41)	56.7	Very low	Small evidence base; moderate heterogeneity; publication bias not reliably assessable.
Hip angular velocity	2	20	0.011 (-0.822, 0.843)	NE	74.3	Very low	Very small evidence base; wide CI; inconsistent effects; publication bias not assessable.
Knee angular velocity	3	33	-0.337 (-0.917, 0.242)	(-6.80, 6.12)	65.2	Very low	Small evidence base; wide CI and extremely wide PI; publication bias not assessable.
Ankle angular velocity	2	20	-0.443 (-0.983, 0.097)	NE	35.8	Very low	Very small evidence base; wide CI; publication bias not assessable.

Note. Negative Hedges' g values indicate poorer post-fatigue performance. PI, prediction interval; GRADE, Grading of Recommendations Assessment, Development and Evaluation; NE, not estimable because fewer than three studies were available.

Supplementary Table S9. Domain-level GRADE judgments for each meta-analysed outcome

Outcome	Risk of bias	Inconsistency	Indirectness	Imprecision	Publication bias	Final certainty
Ball velocity	Serious concern	Serious concern	No serious concern	No serious concern	No serious concern	Low
Kicking accuracy	Serious concern	Serious concern	No serious concern	No serious concern	No serious concern	Low
Foot velocity	Serious concern	Serious concern	No serious concern	Serious concern	Not reliably assessable	Very low
Hip angular velocity	Serious concern	Serious concern	No serious concern	Very serious concern	Not assessable	Very low
Knee angular velocity	Serious concern	Serious concern	No serious concern	Very serious concern	Not assessable	Very low
Ankle angular velocity	Serious concern	No serious to serious concern	No serious concern	Very serious concern	Not assessable	Very low

Note. Domain judgments were made using the GRADE framework. The final certainty rating reflects the overall judgement for each outcome and was not determined by mechanically summing domain concerns. Publication bias was judged as not reliably assessable when fewer than 10 studies were available.

Figure S1. Study-level risk-of-bias assessment using RoBANS-2

	D1	D2	D3	D4	D5	D6	D7	D8
Abt et al. (1998)	+	X	X	+	+	+	−	−
Ali et al. (2007)	+	X	X	+	X	+	−	−
Alkhawaldeh (2022)	+	+	X	+	+	+	−	−
Apriantono (2006)	+	+	X	+	+	+	−	−
Béliard (2019)	+	+	X	+	+	+	−	−
Cariolo (2019)	+	+	X	+	+	+	−	−
Carstensen (2024)	+	+	+	+	+	+	X	−
De Pablo (2024)	+	+	+	+	+	+	−	−
Draganidis (2013)	+	+	+	+	+	+	−	−
Ferraz (2019)	+	+	X	+	+	+	−	−
Ferraz (2012)	+	+	X	−	+	+	−	−
Ferraz (2017)	+	+	X	+	+	+	−	−
Gaspar (2019)	+	+	X	+	X	+	−	−
Gharbi (2017)	+	+	+	+	X	+	−	−
Gopalakrishnan (2024)	+	+	X	+	+	+	−	−
Greig (2018)	+	+	X	+	+	+	−	−
Izquierdo (2020)	+	+	X	X	−	+	−	−
Juárez (2011)	+	+	X	+	+	+	−	−
Katis (2014)	+	+	+	+	+	+	−	−
Katis (2017)	+	+	+	+	+	+	−	−
Kellis (2006)	+	+	+	+	+	+	−	−
Maly (2018)	+	+	X	+	X	+	−	−
McMorris (2000)	+	X	X	+	X	+	−	−
Mor (2022)	+	+	X	+	+	+	−	−
Owen (2013)	+	+	+	+	X	+	−	−
Ozimek (2022)	+	+	X	+	X	+	−	−
Radman (2016)	+	+	+	+	+	+	−	−
Russell (2011)	+	+	+	+	X	+	−	−
Russell (2012)	+	+	+	+	X	+	−	−
Sánchez-Sánchez (2014)	+	+	X	+	+	+	−	−
Stevenson (2017)	+	+	+	+	X	+	−	−
Stone & Oliver (2009)	+	+	+	+	X	+	−	−
Torreblanca-Martínez (2017)	+	+	+	+	+	+	−	−
Vieira (2023)	+	+	+	+	+	+	−	+
Yasar (2025)	+	+	X	+	X	+	−	−

Domains:
D1: Comparability of the target group.
D2: Target group selection.
D3: Confounders.
D4: Measurement of exposure.
D5: Blinding of assessors.
D6: Outcome assessment.
D7: Incomplete outcome data.
D8: Selective outcome reporting.

Judgement
High risk (X)
Unclear (−)
Low risk (+)
